# Global resurgence of norovirus GII.17[P17] accompanies shifting phylogeographic connectivity patterns: surveillance in Jiangsu, 2023–2025

**DOI:** 10.3389/fmicb.2026.1861818

**Published:** 2026-10-05

**Authors:** Chuchu Li, Jinbo Xiao, Jing Ai, Yuanfang Qin, Ke Xu, Liguo Zhu, Shenjiao Wang, Jianguang Fu

**Affiliations:** 1 Key Laboratory of Enteric Pathogenic Microbiology, National Health Commission, Jiangsu Provincial Center for Disease Control and Prevention, Nanjing, China; 2 WHO WPRO Regional Polio Reference Laboratory, NHC Key Laboratory for Biosafety, NHC Key Laboratory for Medical Virology, National Institute for Viral Disease Control and Prevention, Chinese Center for Disease Control and Prevention, Beijing, China

**Keywords:** GII.17[P17], norovirus, outbreaks, phylogenetic analysis, spatiotemporal analysis, VP1 mutation

## Abstract

**Background:**

Norovirus GII.17[P17] has re-emerged and has been circulating globally since 2023. In Jiangsu, China, we found that its proportion increased from 2024 onward. Using local surveillance data combined with global sequences, we reconstructed the global spatiotemporal dynamics of GII.17[P17] and explored its evolution and molecular mechanisms.

**Methods:**

Samples were subjected to whole-genome sequencing using next-generation sequencing. We used bioinformatics tools to analyze the full viral protein 1 sequences for molecular evolution, spatiotemporal phylogeographic associations, and amino acid changes.

**Results:**

From October 2024 to May 2025, 209 GII.17[P17] outbreaks were reported (approximately 26 per month), while only 23 outbreaks occurred in the full year of 2023 (fewer than 2 per month). In sporadic case surveillance, the proportion of GII.17[P17] increased from 9.6% in 2023 to 62.4% in January–May 2025. The maximum clade credibility tree showed that GII.17[P17] clustered into two large clusters. Most samples from 2024 to 2025 belonged to Lineage 3 of Cluster II. In the phylogenetic network and minimum spanning tree, we identified three core hub strains. Inferred phylogeographic connectivity patterns suggest a shift in the primary centers of viral lineage dispersal from East Asia (Cluster I, 2014–2016) to Northwest Europe (Cluster II, 2021–2025). Molecular evolutionary analysis revealed that the current dominant lineage (Lineage 3, Cluster II) exhibited an evolutionary rate of 4.126 × 10^−3^ substitutions/site/year (95% HPD: 3.27–4.98 × 10^−3^), substantially higher than the earlier Cluster I lineages (1.798 × 10^−3^; 95% HPD: 1.41–2.19 × 10^−3^; posterior probability >0.99; 95% HPD intervals non-overlapping).

**Conclusion:**

Inferred phylogeographic connectivity patterns indicate that the primary centers of GII.17[P17] lineage dispersal have shifted from East Asia (2014–2016) to Northwest Europe (2021–2025). The current dominant lineage (Lineage 3, Cluster II) exhibits an accelerated evolutionary rate, approximately 2.3-fold faster than Cluster I lineages, and carries lineage-specific amino acid substitutions in antigenic epitopes temporally associated with its global expansion. These findings highlight the value of sustained genomic surveillance in regions with high inferred phylogeographic connectivity for monitoring norovirus evolution.

## 1 Introduction

Norovirus (NoV) is a leading cause of acute gastroenteritis (Desselberger and Goodfellow, 2014). It is estimated to cause more than 200,000 deaths annually worldwide across all ages (Bartsch et al., 2016). Noroviruses belong to the family *Caliciviridae* and are non-enveloped, single-stranded, positive-sense RNA viruses. Current classification is based on the viral protein 1 (VP1) and RNA-dependent RNA polymerase (RdRp) sequences. This scheme recognizes 10 genogroups (GI–GX) and at least 48 genotypes (Chhabra et al., 2019, 2020).

The GII.4 genotype has remained the dominant global epidemic strain since 1995. During the winter of 2014–2015, the GII.17[P17] Kawasaki 2014 variant spread rapidly across multiple East Asian countries. It became the locally dominant strain (Fu et al., 2015; Matsushima et al., 2015). Following the emergence of GII.2[P16] recombinant strains and new GII.4 variants in 2016, GII.17[P17] was quickly replaced. It has since circulated at low intensity (Ao et al., 2017; Lu et al., 2017; Xue et al., 2019). Since 2023, GII.17[P17] has re-emerged with increased activity. This newly emerged variant has expanded both its geographical reach and epidemiological intensity. It is now detected in multiple countries worldwide. In several regions, it has overtaken other strains as the dominant epidemic strain (Chhabra et al., 2024; Gomes et al., 2025; Novazzi et al., 2026; Zheng et al., 2026).

While recent studies have characterized the global diversification of GII.17 using maximum likelihood phylogenetics (Tohma et al., 2025), formal Bayesian phylogeographic inference with statistical support for specific migration routes has not been performed. A fundamental question remains unanswered: what evolutionary changes enabled the global resurgence of GII.17[P17] since 2023, and how have its transmission dynamics shifted? To address this, we employed a sentinel-to-global framework. First, we used continuous sentinel surveillance in Jiangsu Province, China (2017–2025, three parallel systems: outpatient, inpatient, and outbreak) to detect the resurgence signal. Second, we contextualized this local signal against publicly available global GII.17[P17] VP1 sequences to reconstruct the global spatiotemporal spread using Bayesian phylogeography. Third, we characterized the molecular changes in the dominant lineage through codon-level selection pressure analysis, systematic mutation classification, and structural modeling. Our findings may inform future surveillance strategies targeting key connectivity hubs and provide candidate mutations for experimental validation.

## 2 Materials and methods

### 2.1 Norovirus surveillance

Norovirus surveillance data from January 2017 to May 2025 in Jiangsu Province were included in this study. All cases were laboratory-confirmed. A norovirus infection case was defined as ≥ 3 episodes of loose or watery stools and/or ≥ 1 episode of vomiting within 24 h, and samples (such as feces, anal swabs, or vomitus) tested positive for norovirus nucleic acid. A norovirus outbreak was defined as ≥ 5 epidemiologically linked cases within a 3-day period, with at least 2 cases confirmed by nucleic acid testing. During outbreak investigations, all suspected cases with gastrointestinal symptoms were tested. Outbreak surveillance covered all 13 municipalities in Jiangsu. Outpatient and inpatient surveillance covered five municipalities, representing the northern, central, and southern parts of Jiangsu to ensure geographic representativeness. Real-time reverse transcription-polymerase chain reaction (RT-qPCR) was performed locally, and positive samples were sent to the Jiangsu Provincial Center for Disease Control and Prevention for confirmation.

### 2.2 Nucleic acid extraction and preliminary genotyping

For nucleic acid extraction, anal swabs were processed directly, while fecal samples were first prepared as 10% suspensions. Extraction was carried out with the Tianlong Virus DNA/RNA Extraction Kit (T324, Tianlong, China) on a Gene Rotex 96 automatic extractor (Gene Rotex 96, Tianlong, China). Extracted nucleic acids were either used immediately or stored at −80 °C. Norovirus genotyping was conducted using a conventional RT-PCR method with the HiScript II One Step RT-PCR Kit (P611-01, Vazyme, China). For the GI group, the genotyping primers were MON432 and G1SKR; for the GII group, the primers were MON431 and G2SKR (Chhabra et al., 2021). Preliminary genotype determination was performed using the online Norovirus Genotyping Tool (https://www.rivm.nl/mpf/typingtool/norovirus/).

### 2.3 Whole-genome sequencing (WGS)

A total of 144 GII.17[P17] norovirus strains from 2023 to 2025 were selected for further whole-genome sequencing (WGS) (14 strains in 2023, 116 strains in 2024, and 14 strains in 2025). Amplification was performed with the MicroFuture Norovirus High-Throughput Sequencing Capture Kit (v-090417, MicroFuture, China). Libraries were prepared using the Illumina Nextera XT DNA Library Preparation Kit (15032785, Illumina, USA). Sequencing was performed on an Illumina MiniSeq instrument with the MiniSeq High Output Reagent Kit (15073755, Illumina, USA). Raw sequence data were processed for quality control and assembly using the CLC Genomics Workbench v21.0.

Using complete VP1 sequences of GII.17[P17] strains from this study as queries, BLAST searches against GenBank retrieved 850 VP1 sequences labeled as GII.17. A preliminary maximum-likelihood (ML) tree was constructed in MEGA. Of the 850 initial sequences, 812 could be unambiguously assigned to Cluster I (*n* = 715) or Cluster II (*n* = 97) of GII.17[P17] based on the ML tree topology. The remaining 38 non-GII.P17 sequences formed a separate cluster. For minor lineages (e.g., Lineage 1 of Cluster I), most sequences were retained; only highly similar sequences from the same country were removed. Sequences within the two major clusters (Cluster I and Cluster II) were clustered at ≥99% nucleotide identity using Vsearch v2.22.1: vsearch --cluster_fast VP1_715.fasta --id 0.99 --iddef 0 --centroids VP1_168_centroids.fasta --uc clusters.uc. The --iddef 0 flag uses the most conservative identity definition (matching columns)/(alignment length). Within each cluster, the longest sequence was retained as centroid. 168 representative GenBank sequences were obtained. 24 Jiangsu study strains were added, selected to maximize temporal coverage (2023–2025) and lineage diversity. Rarefaction analysis confirmed that the 24 selected strains effectively captured the full phylogenetic branch-length diversity represented by the 144-strain Jiangsu set. The remaining 120 strains (>99% identical to retained representatives) were excluded to avoid inflating local representation without adding phylogenetic information. Final dataset was 192 sequences (168 GenBank + 24 Jiangsu). Supplementary Tables 1, 2 and Supplementary Figure 1 show regional and temporal composition before and after filtering.

RDP4 recombination analysis (nine methods, Bonferroni *p* < 0.05) on 192 VP1 sequences detected one event, supported by only 2/9 methods and flagged as potentially non-recombinogenic. No meaningful recombination was found in VP1.

### 2.4 Phylodynamics analysis

The optimal nucleotide substitution model for the 192 VP1 region sequences was determined to be GTR + I + G using ModelFinder v1.6.12. Temporal signal strength in the sequence set was evaluated using TempEst v1.5.3 (correlation = 0.899, *R*^2^ = 0.808). No regression outliers were identified in any dataset. The BEAST control file was generated using BEAUti (v1.8.4). Path sampling and stepping-stone sampling compared 12 model combinations (3 clock models × 4 tree priors) across four datasets. Uncorrelated relaxed clock (lognormal) + Bayesian Skyline (Drummond et al., 2005) was consistently the best model (ΔlogML = 8–17 vs. next-best; Supplementary Table 3). The Markov Chain Monte Carlo (MCMC) chain length was set to 1 × 10^9^ steps. BEAST (v1.8.4) was used to run the control file. Tracer (v1.7.2) was employed to check the effective sample size (ESS > 200 was considered to indicate parameter convergence) of each parameter, and the software was also used to generate skyline plots. A burn-in of 10% was applied to all BEAST runs. Post-burn-in trees and parameter traces were used for all downstream analyses. Sampling frequency: 100,000 steps per sample (10,000 posterior samples per run). Priors were BEAUti defaults. Trace plots for key parameters were inspected in Tracer v1.7.2, and all showed good mixing without trends. Complete trace plots and posterior distributions for all parameters are available in the deposited log files, which can be accessed as Supplementary Data via the following link: [https://figshare.com/s/545e085c25b1e8183a2a]. TreeAnnotator (v1.8.4) was used to generate the Maximum Clade Credibility (MCC) tree file, which was subsequently visualized and annotated using FigTree (v1.4.4).

### 2.5 Minimum spanning tree analysis

Based on the generated MCC tree, sequences from GII.17 strains that did not carry the P17 polymerase type were subsequently excluded, retaining only GII.17[P17] strains for downstream analysis. Ultimately, 169 VP1 region sequences spanning two GII.17[P17] clusters (24 study strains and 145 reference strains) were obtained. A phylogenetic network and a minimum spanning tree (MST) for the 169 VP1 sequences were generated using Python. The MST was constructed from a combined distance matrix: combined_distance = base_distance × 0.7 + length_penalty × 0.2 + gc_difference × 0.1. The base genetic distance (p-distance) captures nucleotide substitutions as the primary signal of genetic divergence; the length penalty accounts for small insertions/deletions in the VP1 P2 domain documented in GII.17; and the GC difference term reflects GC-biased mutational processes recognized in RNA virus evolution (Simmonds, 2020). In this dataset (169 VP1 sequences, all ~1,629 bp), length is invariant (SD = 0 bp) and GC content is highly conserved (46.5–48.6%, SD = 0.56%), such that the length and GC components each contribute <0.1% to the combined distance matrix. Hub identification is therefore driven almost entirely by the base genetic distance. Nodes with higher degree centrality were rendered with larger radii and positioned closer to the network center by the Kamada-Kawai layout algorithm. Edge colors represent pairwise combined genetic distance on a continuous viridis scale (blue = low, yellow = high). The MST was generated based on the distance matrix using the Kamada-Kawai layout.

### 2.6 Phylogeographic analysis

Phylogeographic analysis was performed on the 169 GII.17 sequences, including 84 sequences from Cluster I (six study strains, 78 reference strains) and 85 sequences from Cluster II (18 study strains, 67 reference strains). Directionality of viral migration was inferred from Markov jump counts along the MCC tree (Minin and Suchard, 2008). Locations with net positive jump counts were classified as inferred sources (exporters); those with net negative counts as inferred destinations (importers). For all three datasets (169 total sequences, 84 from Cluster I, and 85 from Cluster II), the optimal nucleotide substitution model was GTR + I + G, and the combination of tree prior and molecular clock model was Bayesian Skyline tree prior and Uncorrelated relaxed clock (log-normal). The chain length for the 169 sequences was set to 1 × 10^9^ steps, whereas for Cluster I and Cluster II, it was set to 2 × 10^8^ steps. SpreaD3 software was used to calculate the migration rates, Bayes factors (BFs), and posterior probabilities (PPs) between different regions. Bayesian Stochastic Search Variable Selection (BSSVS) with symmetric discrete diffusion was used to infer migration rates between discrete geographic states. The standard BSSVS Poisson prior (mean = ln 2 ≈ 0.693, equivalent to 50% prior probability that any individual rate is zero) was applied (Lemey et al., 2009). BFs were interpreted using a five-tier framework adopted from Yang et al. (2025): 3 <BF ≤ 10 (marginal support), 10 <BF ≤ 30 (moderate), 30 <BF ≤ 100 (strong), 100 <BF ≤ 500 (very strong), BF > 500 (decisive). Migration routes were considered supported when meeting the dual criterion of BF > 3 and posterior probability PP > 0.5 for the ancestral state trait. In practice, all routes meeting both criteria had BF ≥ 5, making BF = 5 the natural operational lower bound of the marginal tier. Complete BF matrices including non-significant routes are provided in Supplementary Table 4.

To assess robustness of phylogeographic inferences to sampling bias, sensitivity analyses used two orthogonal subsampling strategies applied to 812 sequences assigned to Cluster I (*n* = 715, 2012–2024) and Cluster II (*n* = 97, 2014–2025). Countries were aggregated into seven continental regions (Supplementary Table 1). Geographically balanced (BAL): CI caps 15/10/6, CII caps 8/5/3, yielding 232/180/128 sequences. Stratified random (RND): CI 120/90/60, CII 60/45/30 (2:1 ratio, seed = 42), yielding 180/135/90 sequences. ModelFinder was applied independently to each of the 18 subsampled alignments. The optimal nucleotide substitution models were TNe + G4 for 16 of the 18 alignments, TN + G4 for the BAL-Mild and BAL-Moderate datasets, and GTR + G4 for the RND-Large dataset. A total of 18 independent BEAST MCMC runs were performed, each with 6 × 10^8^ steps, under the GTR + I + G substitution model with a Bayesian Skyline tree prior and an uncorrelated log-normal relaxed clock. Adjusted Bayes factors (BF_adj) via tip-state-swap corrected for sampling-imbalance inflation (Gámbaro et al., 2025). Complete matrices in Supplementary Table 4. All BEAST XML control files and analysis scripts associated with this study have been deposited in the Figshare repository and are available as Supplementary Data via the following link: [https://figshare.com/s/545e085c25b1e8183a2a].

### 2.7 VP1 amino acid mutation analysis

BioEdit software was used to identify key amino acid mutation sites across different branches, including antigenic sites, receptor-binding sites (RBS), and unique mutation sites (Tan and Jiang, 2010; Tohma et al., 2025). The three-dimensional (3D) protein structure of the protruding (P) domain of norovirus GII.17 Kawasaki323 (PDB ID: 5F4M) was downloaded from the Protein Data Bank (PDB) website (https://www.rcsb.org/). Preliminary homology model construction and quality assessment were performed using the SWISS-MODEL online tool (Waterhouse et al., 2018) with the GII.17 Kawasaki323 P domain structure as template. The target shares 95.39% amino acid sequence identity with the template over 96% coverage (residues 4–307), substantially above the 90% threshold for reliable homology modeling. Model quality metrics were: Global Model Quality Estimation (GMQE) = 0.90, Qualitative Model Energy ANalysis with Distance Constraints (QMEANDisCo) Global = 0.85 ± 0.05. Final model visualization and rendering were performed in ChimeraX v1.7. Codon-level selection pressure on VP1 was assessed using three complementary methods in HyPhy v2.5: MEME (episodic diversifying selection), FEL (pervasive selection), and SLAC (conservative verification). Analyses on 192 VP1 sequences (543 codons) aligned to AB983218 (Kawasaki323/2014/JPN).

## 3 Results

### 3.1 Increased epidemic intensity

Prior to January 2023, the overall number of norovirus GII.17[P17] outbreaks was relatively low (Figure 1A). Only 14 outbreaks were reported in total between January 2017 and December 2022. Thereafter, outbreaks rose to 23 from February to December 2023 (fewer than 2 per month) and 14 from January to September 2024 (~1.6 per month), then sharply to 209 between October 2024 and May 2025 (~26 per month). From October 2024 onward, both the total number of outbreaks and the relative proportion of this genotype indicated that it had become the dominant strain in Jiangsu Province while other genotypes did not show a corresponding increase.

**Figure 1:**
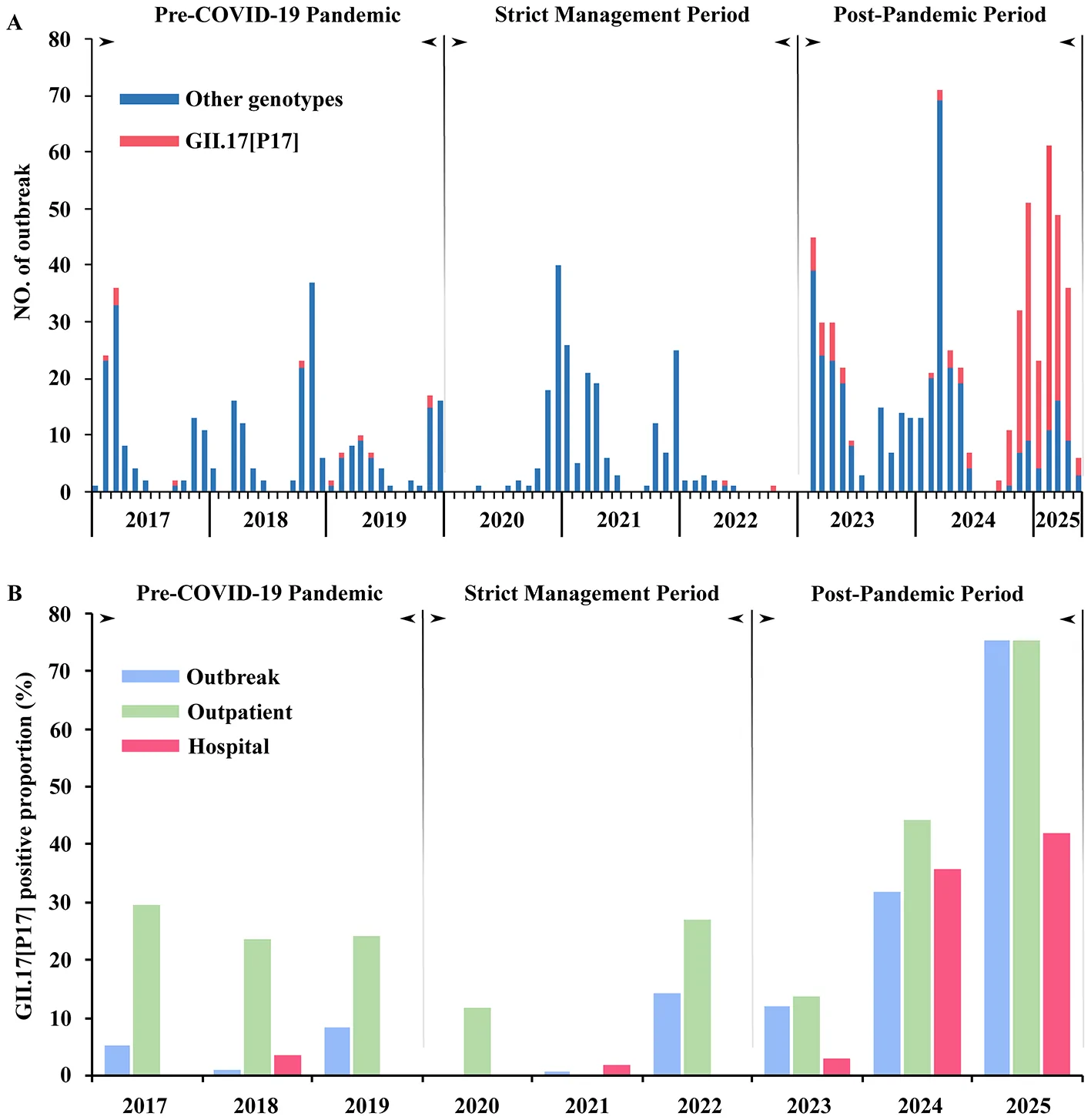
Emergence of GII.17[P17] norovirus associated with acute gastroenteritis before, during, and after the COVID-19 pandemic, Jiangsu, China, January 2017–May 2025. **(A)** Number of laboratory-confirmed monthly GII.17[P17] norovirus outbreaks. **(B)** Distribution of GII.17[P17] among typed norovirus-positive specimens from outbreak, outpatient, and hospitalized cases. The strict management period of the COVID-19 pandemic in Jiangsu spanned from January 2020 to December 2022 (shaded region).

Between 2017 and 2022, GII.17[P17] was detected mostly in outpatient settings (Figure 1B). Except for 2021, when we did not detect any GII.17[P17]-positive cases, the proportion of GII.17[P17] among norovirus RT-qPCR screening-positive specimens in other years ranged from 11.8% to 29.4%. In 2023, the proportions in outpatient settings and outbreaks were close: 13.6% vs. 11.9%. From 2017 to 2023, the proportion among hospitalized cases remained below 5%. Starting in 2024 and continuing into 2025, the proportion increased sharply across all three settings: outbreaks, outpatient clinics, and hospital patients. The overall proportion among norovirus RT-qPCR screening-positive specimens reached 35.1% in 2024 and climbed further to 62.4% in January–May 2025.

Genotype replacement dynamics over the 9-year surveillance period are shown in Supplementary Figure 2. Before 2024, GII.17[P17] was a minor contributor at low levels (<16% across all systems, 2017–2019), co-circulating with GII.4 Sydney[P31], GII.4[P16], GII.2[P16], and GII.3[P12]. In 2020–2021, during the strict COVID-19 non-pharmaceutical intervention period, GII.17[P17] nearly disappeared (0–3.3%). The resurgence began in 2024, when GII.17[P17] rose sharply to 35.1% of all typed cases, and accelerated further to 62.4% in 2025 (January–May), coinciding with the decline of all other genotypes. The quarterly outbreak timeline (2017–2025) shows that the 2024–2025 outbreak wave is the largest in the entire surveillance period. The increase in GII.17[P17] detection occurred synchronously across three independent surveillance systems—outpatient sentinel (13.6% to 62.5%), inpatient (2.9% to 41.1%), and outbreak (12.0% to 75.4%)—representing fold increases of 4.6 ×, 14.2 ×, and 6.3 × respectively from 2023 to 2025 (Supplementary Figure 3).

### 3.2 Time-scaled phylogenetic analysis

An MCC tree was constructed from the 192 full-length VP1 sequences of GII.17 strains, with different colors representing distinct evolutionary lineages (Figure 2A). Within this tree, strains with a non-GII.P17 RdRp region (GII.17[P3], GII.17[P13], GII.17[P16], and GII.17[P31]) were included solely for outgroup rooting and clustered together into a single large clade at the top of the tree. This clade was further subdivided into two clusters: Cluster I contained two lineages (Lineage 1 and Lineage 2), and Cluster II contained three lineages (Lineage 1–Lineage 3).

**Figure 2:**
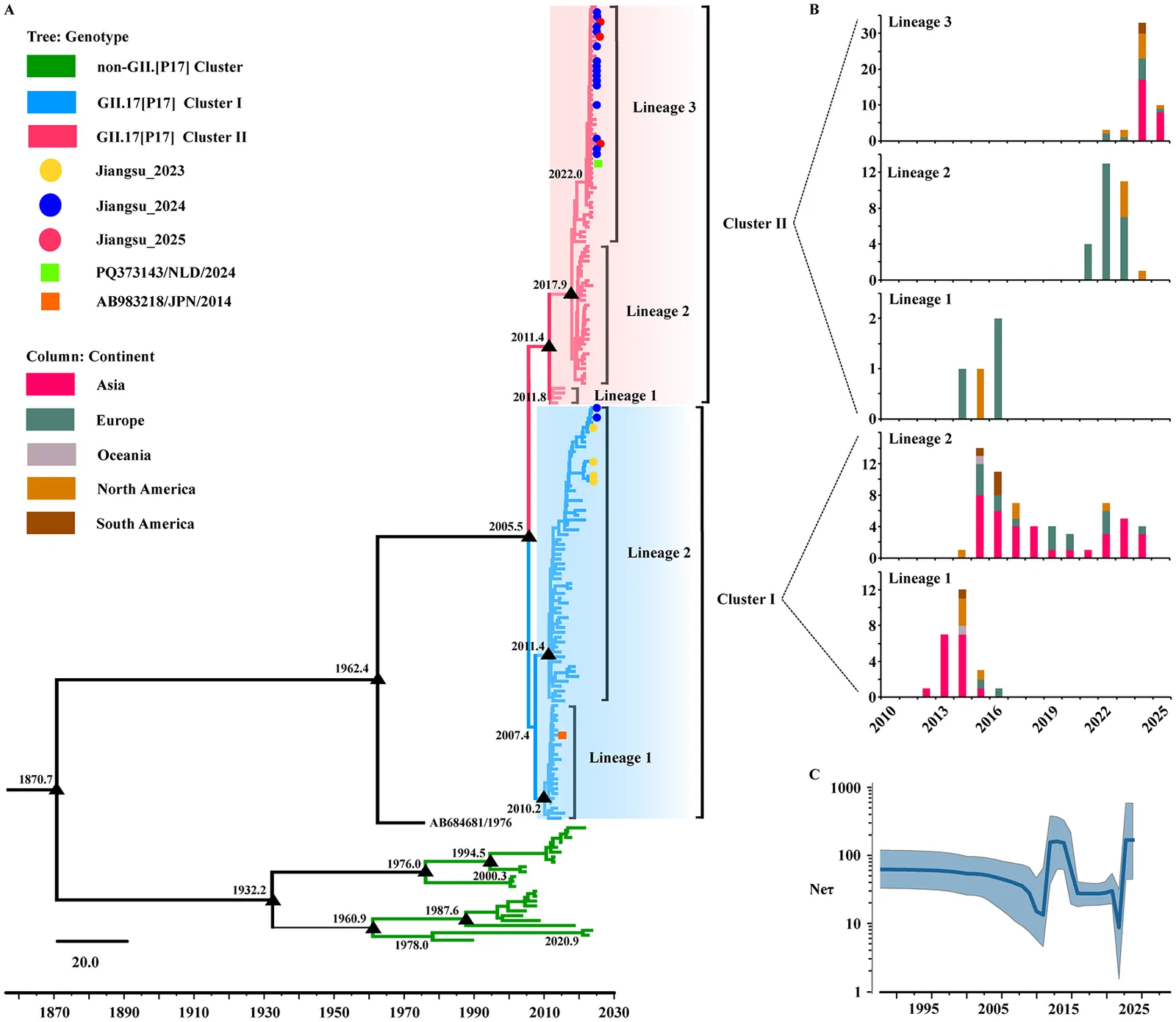
Bayesian time-scaled phylogeny and demographic history of GII.17 strains. **(A)** Maximum clade credibility (MCC) tree reconstructed from 192 full VP1 nucleotide sequences. Branches are colored by Cluster: Cluster I (blue), Cluster II (red), and non-GII.P17 Cluster (green). Filled circles indicate Jiangsu study strains, color-coded by sampling year: yellow (2023), blue (2024), red (2025). The 2024 Dutch hub strain (PQ373143/NLD/2024) is marked with a green square. The GII.17 Kawasaki323 reference strain (AB983218/2014/JPN) is annotated. Black triangles at nodes indicate posterior probability > 0.90. Scale bar is in years. **(B)** Geographic distribution of GII.17 strains across lineages within Cluster I and Cluster II. Continents are color-coded: Asia (red), Europe (dark green), North America (orange yellow), South America (dark brown), Oceania (grayish purple). **(C)** Bayesian skyline plot of GII.17 VP1 sequences. The y-axis represents effective population size (Neτ), and the x-axis denotes time in years. The solid blue line represents the median posterior estimate; the blue shading indicates the 95% highest posterior density (HPD) interval.

Lineage 1 of Cluster I comprised reference sequences from 2012 to 2016, mainly from Asia and North America (Figure 2B). Lineage 2 of Cluster I comprised reference sequences from 2014 to 2024, mainly from Asia and Europe; a small portion of the study strains were located within this branch. Lineage 1 of Cluster II comprised reference sequences from 2014 to 2016, primarily from Europe and North America. Lineage 2 of Cluster II comprised reference sequences from 2021 to 2024, similar to Lineage 1, which was mainly from Europe and North America. Lineage 3 of Cluster II comprised reference sequences from 2022 to 2025. Among these, the 2022–2023 reference sequences were mainly from Europe and North America; the 2024 sequences were from Asia, Europe, and North America; and the 2025 reference sequences were primarily from Asia. Most of the study strains were situated within the 2024–2025 branch.

The effective population size reflects the level of genetic diversity accumulated by GII.17[P17] over time (Figure 2C). The Bayesian skyline plot suggested two apparent population bottlenecks around 2012 and 2022, followed by rapid expansions. However, the 95% highest probability density (HPD) intervals around these periods are wide, reflecting sparse early sampling, and the apparent bottlenecks may partially reflect sampling artifacts rather than definitive demographic events. After the first expansion, genetic diversity plateaued in 2014–2015. The curve appears to begin plateauing in the most recent period, but inference regarding the second half of 2025 is precluded by the sampling cutoff (May 2025).

### 3.3 Hub strains within the phylogenetic tree

To identify strains occupying highly connected positions in the genetic distance network, we analyzed the VP1 sequences of GII.17 strains carrying the GII.P17 RdRp type based on their mutual genetic distance relationships. Phylogenetic network and minimum spanning tree (MST) analysis of the 169 GII.17[P17] VP1 sequences identified three core hub strains occupying key connecting positions within the network (Figure 3). Three strains (KP864102/CHN/2015, OM480530/CHN/2020, and PQ373143/NLD/2024) were consistently identified across multiple validation approaches. The robustness of this identification was supported by three lines of evidence: (i) top degree centrality in 100% of 38 parameter combinations, (ii) support from ≥2 centrality metrics (degree, betweenness, PageRank), and (iii) >50% bootstrap recovery (200 iterations; Supplementary Figures 4–7). Two Asian hub strains (KP864102 and OM480530) belonged to Cluster I, while the European hub strain (PQ373143) emerged within Cluster II, suggesting a temporal shift in network centrality from East Asia to Northwest Europe. These three nodes were also marked on the MST (right panel), with dashed lines connecting the same hub nodes in both figures. In the MST, the two Asian strains fell in Lineage 2 of Cluster I, and the European strain PQ373143/NLD/2024 was in Lineage 3 of Cluster II (Figure 3B).

**Figure 3:**
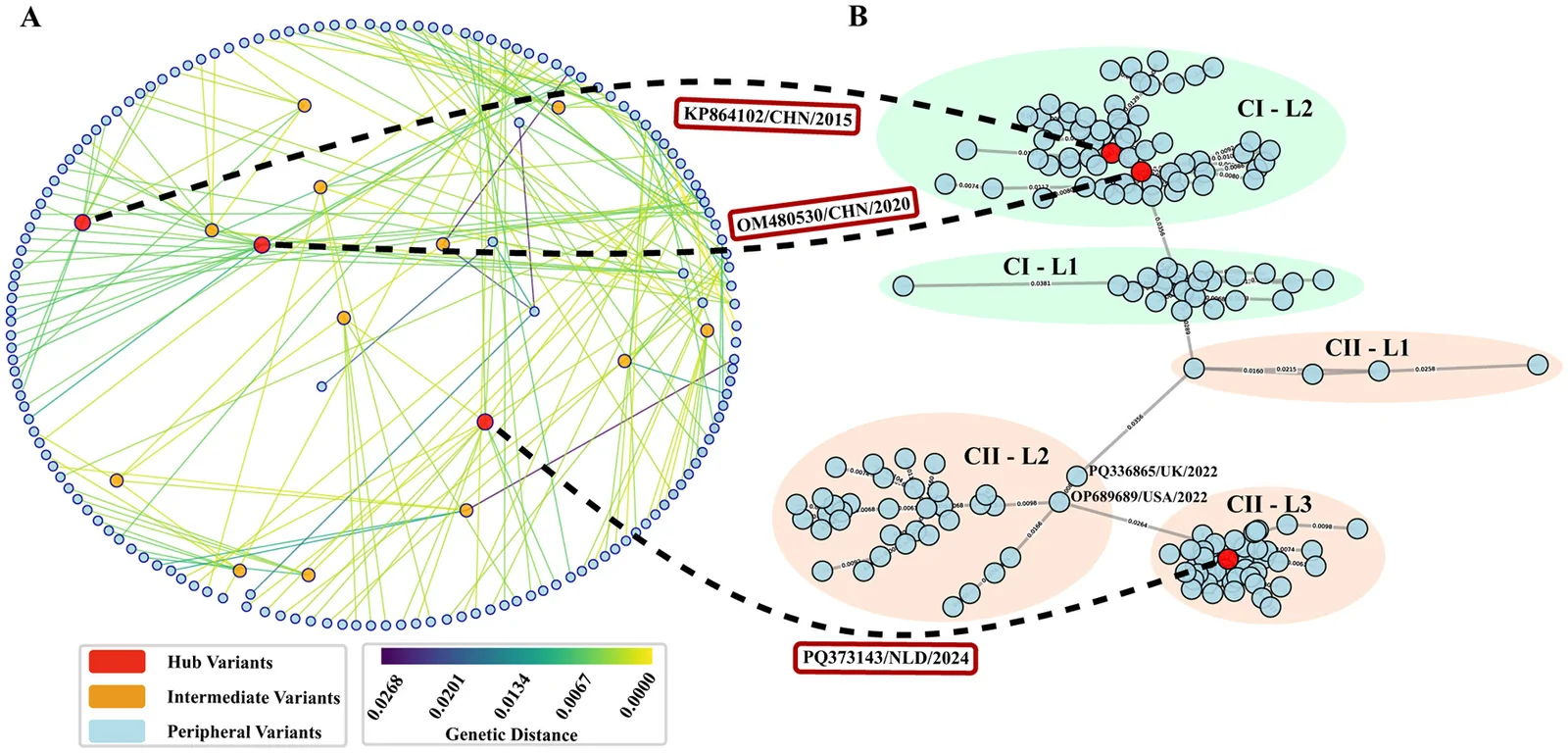
Phylogenetic network and minimum spanning tree (MST) of 169 GII.17[P17] VP1 sequences. **(A)** Phylogenetic network with nodes sized by degree centrality (hub: 250 pts; intermediate: 150 pts; peripheral: 25–50 pts) and colored by variant classification: red, three core hub variants validated by multi-criteria analysis; yellow, intermediate variants; blue, peripheral variants. Edge colors represent pairwise combined genetic distance (viridis scale: blue = low, yellow = high). Inset: degree distribution with the three validated hubs highlighted. **(B)** Minimum spanning tree with the three core hub variants connected by dashed lines and annotated with strain names.

### 3.4 Global spatial transmission routes

The global spatial phylogeographic associations inferred from the total 169 VP1 sequences revealed 19 migration routes. All 19 routes in Figure 4A have BF > 10, exceeding the marginal tier. Among these, the Netherlands and the United Kingdom (UK) in Europe, and China and Japan in Asia, appeared as highly connected nodes in the inferred network, followed by the United States (USA), Russia, and Argentina as important links. GII.17[P17] exhibited strong intercontinental phylogeographic associations. Highly connected nodes in the inferred network included the Netherlands (Western Europe), which was linked to Russia (Eastern Europe), China (East Asia), the USA (North America), and Argentina (South America), all with BF > 500. The Netherlands had a net positive Markov jump count in the combined dataset, consistent with its inferred role as a predominant source of viral lineage dispersal. Some BF values exceeded 3,000 for associations involving the Netherlands, indicating its high connectivity within the inferred network.

**Figure 4:**
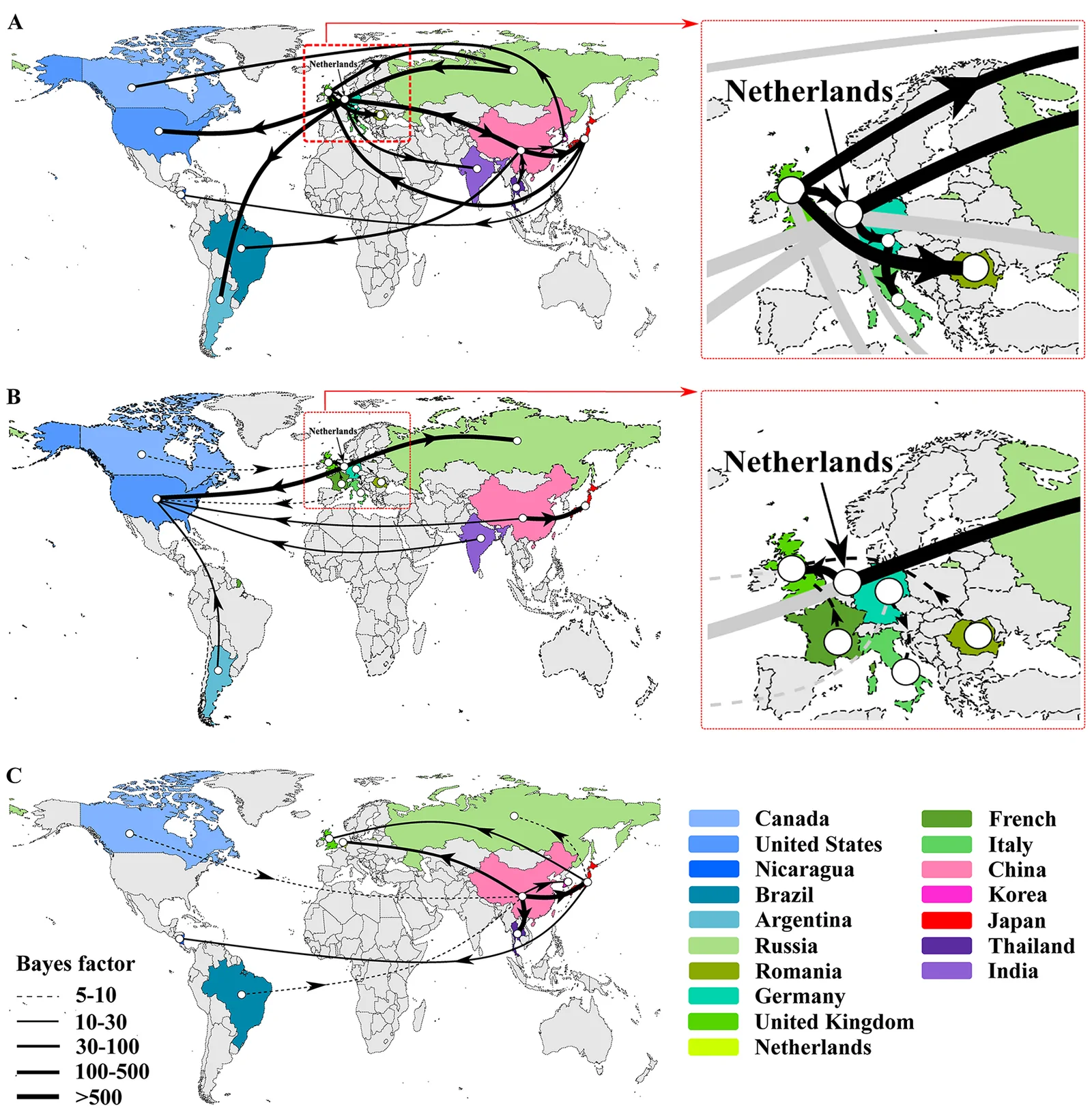
Inferred phylogeographic migration links of GII.17[P17] VP1 sequences at a global scale. Maps displaying statistically supported non-zero migration rates (*BF* > 3, *PP* > 0.5) for **(A)** combined Clusters I and II, **(B)** Cluster II, and **(C)** Cluster I. Arrowheads indicate the predominant direction of inferred migration based on Markov jump counts. Line thickness reflects the strength of statistical support according to the five-tier Bayes factor classification: dashed, 5 <*BF* ≤ 10 (marginal); thin solid, 10 <*BF* ≤ 30 (moderate); medium solid, 30 <*BF* ≤ 100 (strong); thick solid, 100 <*BF* ≤ 500 (very strong); thickest solid, *BF* > 500 (decisive). The red inset shows an enlarged view of Europe: gray routes denote intercontinental links (de-emphasized), while black routes denote intra-European links. Complete Bayes factor, posterior probability, and migration rate values for all pairwise routes are provided in Supplementary Table 4.

Phylogeographic associations inferred from the 85 VP1 sequences of Cluster II identified 12 significant migration routes (*BF* > 5, *PP* > 0.5; Figure 4B). The migration routes of the USA in North America and the UK and the Netherlands in Europe exhibited the highest inferred phylogeographic connectivity in the global spread, with China also being an important link. Phylogeographic inference identified the Netherlands as a net exporter (positive Markov jump count), with statistically supported migration links to Russia in Eastern Europe (BF > 6,000) and the USA in North America (BF > 3,000). A supported migration link also existed between the Netherlands and the UK, with the Netherlands inferred as the net exporter (BF > 90). The USA was involved in five inferred migration links, but functioned primarily as a net importer (negative Markov jump count). A strongly supported migration link existed between China and Japan (BF > 20,000).

Phylogeographic associations inferred from the 84 VP1 sequences of Cluster I identified nine significant migration routes (*BF* > 5, *PP* > 0.5; Figure 4C). China and Japan appeared as the most highly connected nodes, with six associations related to China and four related to Japan. Highly significant associations existed between China and Japan, as well as China and Thailand, with BF values above 1,000. Additionally, in intercontinental associations, a supported route existed between China and the Netherlands (BF > 90).

Two orthogonal subsampling strategies were applied to evaluate the sensitivity of phylogeographic inference to sampling bias. For Cluster II, Northwest Europe consistently emerged as the primary destination under all six strategies. Migration routes from East Asia to NW Europe (BF = 90–14,712) were significant in all six strategies. Under the strictest geographically balanced strategy (BAL-Strict), with the Netherlands limited to three sequences (29 total Cluster II sequences), key routes remained supported: East Asia to NW Europe (BF = 24) and North America to NW Europe (BF = 91).

For Cluster I, BSSVS initially identified Oceania as strongest source region (BF_std = 5–19,237). Comparison of standard and adjusted Bayes factors showed this to be a statistical artifact. Under random subsampling with two Oceania sequences, BF adj declined to 0.5–0.7; under geographically balanced subsampling with nine sequences, BF adj ranged from 30–74, representing a 2- to 5-fold exaggeration by BF std. Oceania was connected only to East Asia, with all other BF adj values below 3. East Asia was confirmed as the true connectivity hub, with BF_adj to all six other regions ranging from 4 to 481. A continental-level comparison of standard and adjusted Bayes factors across all subsampling strategies is provided in Supplementary Table 4 and Figure 8.

### 3.5 VP1 region mutation analysis

The amino acid substitutions at key positions within the VP1 region across different lineages were illustrated, including antigenic epitopes A, D, and E, receptor-binding sites I–III, and other lineage-specific mutations (Figure 5A). This study primarily focused on the distinctive substitutions within Lineages 2 and 3 of Cluster II. At position 372 of antigenic epitope A, Lineage 1 of Cluster II possessed arginine (R), while Lineage 2 exhibited a mixture of R and lysine (K), and Lineage 3 featured only K. At position 395 of antigenic epitope D, for Cluster II, Lineages 1 and 3 both possessed asparagine (N), whereas Lineage 2 exhibited a mixture of N and aspartic acid (D), similar to Cluster I. A similar situation was observed at position 350 of receptor-binding site I, where Lineage 3 of Cluster II featured serine (S), whereas Lineage 2 contained a mixture of S and alanine (A), which was the same as in other lineages. At other sites, such as position 361 in the P2 region, Lineages 1 and 2 of Cluster II contained either K or R, whereas Lineage 3 featured exclusively R. At position 396, Lineage 3 featured glutamine (Q), whereas the other lineages possessed histidine (H). At position 469 in the P1.2 region, Lineage 3 featured glutamic acid (E), whereas the other lineages uniformly possessed D.

**Figure 5:**
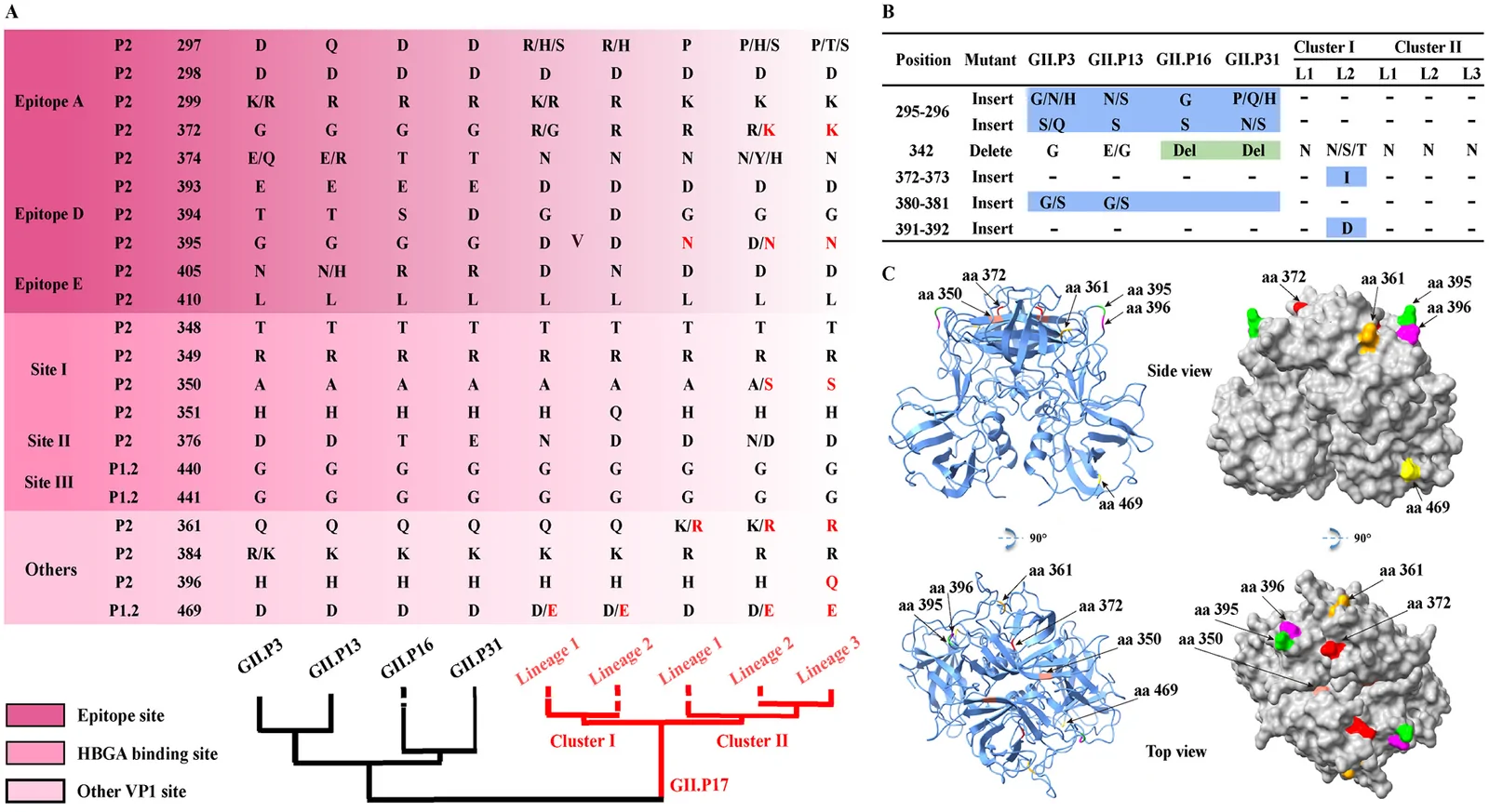
Informative amino acid sites within the GII.17 VP1 protein. Residue numbering in Panels A and B follows the GII.17 Kawasaki323 reference strain (AB983218/2014/JPN; PDB: 5F4M). **(A)** Amino acid substitution sites across phylogenetic lineages. Rows from top to bottom on the left side are: antigenic epitopes A, D, E; histo-blood group antigen (HBGA)-binding sites I–III; other P sub-domain sites. Unique substitutions in Cluster II Lineage 3 are highlighted in red. The simplified phylogenetic tree at the bottom indicates the corresponding lineage relationships. **(B)** Amino acid insertions and deletions in the VP1 region. **(C)** Structural model of the GII.17 P domain (homology model based on PDB: 5F4M; GMQE = 0.90, QMEANDisCo = 0.85 ± 0.05), shown from top and side views. Key residues are annotated with arrows and text labels.

In addition to substitutions, VP1 evolution involved insertions or deletions (Figure 5B). Among these, variants with a non-GII.P17 RdRp region exhibited amino acid insertions between positions 295–296 and 380–381. Lineage 2 of Cluster I had one amino acid inserted between positions 372–373 and 391–392. The GII.17[P16] and GII.17[P31] variant lineages exhibited a deletion at position 342. However, no characteristic insertions or deletions were observed in Cluster II Lineages 2 and 3. Homology modeling was performed using the AB983218/2014/JPN strain as a template (PDB: 5F4M). Homology modeling predicted that these six residues are all surface-exposed. The substitution at position 396 (purple), unique to Lineage 3, was spatially proximal to the substitution at position 395 (green) within antigenic epitope D (Figure 5C).

Of 543 VP1 residues, 101 variable positions across 10 phylogenetic subpopulations were classified into three categories. Category I (Critical Fixed, *n* = 12) comprised positions fixed (≥80%) in ≥1 lineage with support from MEME/FEL selection, convergent evolution in ≥3 branches or multi-lineage fixation in a known functional domain. Category II (Fixed, Lineage-Specific, *n* = 84) comprised positions fixed in at least one lineage but lacking independent functional evidence. Category III (Sporadic/Random, *n* = 5) comprised low-frequency positions (<80%) with no spatiotemporal persistence and no selection signal, consistent with neutral genetic drift. The remaining 442 positions (81.4%) were fully conserved under purifying selection (FEL, *p* < 0.1). A complete per-position frequency matrix is provided in Supplementary Table 5.

Under the three-tier classification, only two Lineage 3 mutations qualified as Critical Fixed: position 372 (R → K, Epitope A), fixed in seven lineages, and position 395 (D → N, Epitope D), fixed in eight lineages. The remaining four mutations (350, A → S RBS-I; 361, Q → R P2; 396, H → Q; 469, D → E P1.2) were classified as Fixed, Lineage-Specific. No positive selection signal was detected by MEME or FEL at these positions, suggesting genetic hitchhiking (Supplementary Table 5). Although position 361 (K361R) was classified as lineage-specific by automated criteria, Tohma et al. (2025) independently demonstrated that this substitution enhances receptor binding and expands host tropism to non-secretor individuals.

MEME identified 15 codons under episodic diversifying selection (*p* < 0.1), with the strongest signals at codon 26 (LRT = 17.77, *p* < 0.001), codon 407 (LRT = 13.04, *p* < 0.001), and codon 530 (LRT = 10.06, *p* = 0.003). Codon 296, located in the P2 hypervariable loop, showed convergent evolution on 10 independent branches (LRT = 10.28, *p* = 0.003), strongly suggestive of immune-driven selection (Lindesmith et al., 2012). Selection sites clustered in three spatially coherent regions (binomial test, *p* < 0.01): P2 hypervariable loop (296, 302), the P2 dimer interface (375, 378, 389), and the P2 C-terminal (407, 429). FEL identified three codons under pervasive positive selection (21, 295, 428). An additional 384 codons (70.7% VP1) were under purifying selection. No positively selected sites were identified by SLAC (Supplementary Table 6).

Nucleotide substitution rates were estimated for the different datasets (Table 1). The mean substitution rate for the 192 sequences was 2.838 × 10^−3^ substitutions/site/year. For the combined 169 sequences in Clusters I and II, the rate was 3.058 × 10^−3^ substitutions/site/year. Cluster I (84 sequences) exhibited a rate of 1.798 × 10^−3^ substitutions/site/year, whereas Cluster II (85 sequences), which included the current variant, showed a rate of 4.126 × 10^−3^ substitutions/site/year.

**Table 1:** Mean rates of nucleotide substitutions per site per year inferred by Bayesian analysis.

Cluster	Number of sequence	Nucleotide substitution rate (10^−3^)	95% HPD (10^−3^)
Cluster I	84	1.798	1.4125–2.1948
Cluster II	85	4.126	3.2715–4.9774

## 4 Discussion

We examined the global migration routes and key hubs of GII.17[P17]. Phylogeographic and molecular evolution analyses showed a synchronized, multi-hub pattern of global spread. The Netherlands, the UK, and the USA exhibited the highest phylogeographic connectivity in the inferred network, serving as highly connected nodes linking international migration routes. Mutations at key amino acid residues in the VP1 region may also contribute to transmission fitness.

The local circulation pattern of GII.17[P17] shifted from sporadic outbreaks before and during the pandemic control periods (2017–2023) to regional dominance immediately after control measures ended (2024–2025). The sharp increase in the proportion of GII.17[P17] among typed norovirus-positive specimens coincided with distinct clinical and epidemiological features. The proportion of outpatient and hospitalized cases has risen sharply since 2024, with hospital admissions lagging behind outpatient detections. This lag may reflect an expanded susceptible population driven by immune evasion, consistent with amino acid substitutions located within or proximal to known antigenic epitopes (Chan et al., 2015).

Surveillance data showed that the overall norovirus epidemic in Jiangsu was significantly impacted during the COVID-19 pandemic. During the strict COVID-19 management period (January 2020–December 2022), the near-complete absence of GII.17[P17] contrasts with continued low-level circulation of other norovirus genotypes. Norovirus is transmitted via the fecal-oral route through contaminated surfaces, food, and water—pathways less affected by respiratory-focused non-pharmaceutical interventions. Low-level circulation can persist in closed institutional settings (schools, nursing homes, hospitals) even during broader community restrictions. The post-pandemic resurgence of GII.17[P17] must be interpreted within a broader context encompassing multiple potential contributing factors beyond viral evolution alone. First, the prolonged period of reduced norovirus circulation during 2020–2022 likely resulted in waning population-level immunity and an expanded susceptible pool, a pattern consistent with immunity debt hypotheses proposed for respiratory viruses during the pandemic (Cohen et al., 2021). This phenomenon may similarly apply to enteric viruses whose circulation was suppressed during the same period. Second, post-pandemic normalization of healthcare access may have contributed to increased case ascertainment. Third, enhanced genomic surveillance capacity established during the pandemic may have improved detection sensitivity. Fourth, the relaxation of international travel restrictions and resumption of population mobility likely facilitated the introduction and spread of new lineages, including Lineage 3. Fifth, stochastic founder effects during the population bottleneck of 2020–2022 may have shaped the genetic composition of re-emerging variants. We therefore interpret the observed resurgence as likely reflecting a confluence of immunological, behavioral, epidemiological, and evolutionary factors, rather than viral evolution alone.

Phylogenetic analysis revealed that GII.17[P17] underwent a rapid lineage replacement within approximately 1 year in Jiangsu. The time-scaled tree placed the 2014 Kawasaki strain together with our 2023 sample strains in Lineage 2 of Cluster I. By 2024, strains had split between Lineage 2 (Cluster I) and Lineage 3 (Cluster II) suggesting a transitional state. By 2025, all the strains belonged to Lineage 3 of Cluster II. These findings indicate that the currently circulating variants form a distinct new cluster. Effective population size analysis indicated a rapid expansion after a bottleneck in 2022, a pattern resembling the early phase of the 2014 Kawasaki outbreak (Matsushima et al., 2019). The increase was larger than that observed in 2014–2015, but formal statistical comparison of effective population size estimates was not performed.

Unlike the 2014 outbreak, during which sustained transmission was concentrated in Asia with sporadic detection on other continents, the current GII.17[P17] epidemic is globally synchronized. It appeared in several Northern Hemisphere countries in late 2023 or early 2024, and reached Argentina in the Southern Hemisphere by June 2024 (Chhabra et al., 2024; Gomes et al., 2025; Li et al., 2026). Based on genetic distance, we identified three hub variants: two from Asia and one from Europe. Although based on only three hub variants and therefore preliminary, both earlier hubs (KP864102/CHN/2015 and OM480530/CHN/2020) were all identified in Asia, whereas the most recent hub (PQ373143/NLD/2024) was from the Netherlands. This temporal distribution is broadly consistent with the cluster-level phylogeographic patterns described above, though this remains an inference based on a limited number of hub variants and further evidence would be needed to confirm. Our 2024–2025 strains belonged to the same lineage as the 2024 Dutch hub variant. In addition, two strains from the USA and the UK (OP689689/USA/2022, PQ336865/UK/2022) were positioned at the evolutionary transition point between Lineage 2 and Lineage 3 of Cluster II. These phylogenetic relationships suggest that multiple regions, including China, the USA, and the UK along with the Netherlands, may act as significant nodes in the global transmission network of GII.17[P17].

The phylogeographic analysis across Clusters I and II showed that the global spread of GII.17[P17] was driven by multiple hubs. The Netherlands exhibited the highest number of inferred phylogeographic connections. While we note the qualitative alignment between this finding and the Netherlands' status as a major international travel hub, this association was not formally tested, and the relative contributions of travel volume, population density, and genomic surveillance intensity cannot be disentangled with the current dataset. Analysis of Cluster I confirmed that its key hubs were China and Japan, consistent with the 2014–2015 East Asian epidemic (Matsushima et al., 2015; Jin et al., 2016). In contrast, Cluster II showed that, in addition to the Netherlands, the UK and USA were involved in multiple routes. This characteristic is highly consistent with our earlier conjecture regarding hub strains. Specifically, there was a clear regional transmission network between the United Kingdom and the Netherlands, as well as several other European countries. As the net importer, the United States was linked to five transmission routes originating in the Netherlands, Germany, Argentina, China, and India. However, in actual transmission events, the roles of net exporters or importers are not absolute, and it is likely that the United States also acted as the net exporter. Many of these countries serve as key connectivity nodes within their continents, characterized by dense populations and well-developed logistics. These features create key routes for fecal-oral diseases to spread locally and cross borders. Similar patterns of high phylogeographic connectivity at international travel hubs have been described for other pathogens (Salami et al., 2020; Shrestha et al., 2020).

VP1 analysis revealed a shift from polymorphism to fixation in the currently dominant lineage. In early lineages of Cluster I and Cluster II (Lineages 1 and 2), multiple amino acid sites were polymorphic, with two or more residues coexisting, whereas in the Lineage 3 these sites quickly became fixed to a single amino acid. This pattern suggests that early parallel evolution eventually pushed GII.17[P17] toward a mutation profile now predominant in the circulating population. Structural modeling predicted that all six unique mutation sites lie on the protein surface, consistent with potential roles in structural stability, antigenic variation, and host interactions. Among these, the evolution at amino acid 361 is temporally associated with the emergence of the dominant lineage. Tohma et al. (2025) demonstrated that K361R enhanced Lewis antigen binding, facilitating infection of non-secretors and expanding the susceptible host range. This mechanism, combining enhanced receptor affinity with antigenic variation, closely resembles the strategy underlying the 2014–2016 GII.17 pandemic. That pandemic relied on the same properties to evade pre-existing herd immunity and establish a transmission advantage (Zuo et al., 2021). Transitional strains at the Cluster II Lineage 1 to Lineage 2 boundary, such as PQ336865/UK/2022, retained lysine (K) at position 361, whereas all Lineage 3 strains carried arginine (R). MST positioning placed this strain at the node marking the lineage transition, and episodic diversifying selection (MEME, p <0.1) was detected during the Lineage 2 to Lineage 3 shift, consistent with refinement of the receptor-binding site through K361R. Phylogenetic data support this mutation's importance for adaptive evolution. Cluster I exhibited an evolutionary rate of 1.798 × 10^−3^ substitutions/site/year, comparable to that reported for early GII.17 strains (Sang and Yang, 2018) and indicative of an evolutionarily conservative pattern. In contrast, Cluster II, which includes the current variants, showed a markedly accelerated rate of 4.126 × 10^−3^ substitutions/site/year. This elevated substitution rate may have enabled the rapid accumulation of advantageous mutations, facilitating immune escape and driving the emergence of new epidemic strains.

Several limitations apply to this study. First, BSSVS infers migration rates among sampled locations, not confirmed transmission events. Second, the inferred network reflects available sequence data; geographically balanced subsampling and BF_adj analyses show that core hub inferences survive equalized geographic representation, though residual surveillance effects cannot be excluded. Third, Africa, the Middle East, and parts of South America and Southeast Asia remain underrepresented, limiting conclusions about these regions. Fourth, travel volume and infrastructure were not formally tested. Fifth, BF_adj reduces Type I errors at the expense of Type II errors under extreme sampling imbalance; in Cluster I, Oceania BF_std values (up to 19,237) were sparse-sampling artifacts (BF_adj <1). Sixth, single MCMC chains were used for primary phylodynamic analyses, mitigated by convergent support across 18 independent sensitivity runs. Seventh, by May 2025, GenBank still did not yet contain complete VP1 sequences of GII.17[P17] from elsewhere in China covering 2024 to 2025. We used Jiangsu sequences as a proxy for China. Including sequences from a wider geographic range in the future would help us assess the generalizability of our conclusions. Finally, the post-pandemic resurgence likely reflects multiple immunological, behavioral, and evolutionary factors whose relative contributions we do not quantify. Despite these caveats, core findings are consistent across geographic balancing, BF adjustment, and subsampling strategies: the shift in inferred connectivity from East Asia (Cluster I) to Northwest Europe (Cluster II), the accelerated evolutionary rate in the current lineage, and convergent amino acid substitutions in antigenic epitopes.

Functional roles of amino acid substitutions predicted by homology modeling and selection analyses require experimental confirmation. K361R is an exception, independently validated by Tohma et al. (2025) to enhance Lewis antigen binding and expand host tropism. We propose prioritized validation targets: receptor-binding assays (position 296, convergent selection across 10 branches), antibody neutralization (positions 372 and 395, Epitopes A and D), and glycan array profiling (P2 hypervariable loop).

## 5 Conclusion

Inferred phylogeographic connectivity patterns indicate that the primary centers of GII.17[P17] lineage dispersal have shifted from East Asia (2014–2016) to Northwest Europe (2021–2025). The Netherlands and the United Kingdom emerged as highly connected nodes in the sampled network for Cluster II, while China and Japan were the principal connectivity hubs in Cluster I; these country-level patterns were robust to geographic balancing across six subsampling strategies. The current dominant GII.17[P17] lineage (Lineage 3, Cluster II) exhibits an accelerated evolutionary rate, approximately 2.3-fold faster than Cluster I lineages. This lineage carries lineage-specific amino acid substitutions in key antigenic epitopes that may contribute to its enhanced spread. Sustained genomic surveillance in regions with high inferred phylogeographic connectivity, combined with experimental characterization of candidate mutations identified here, will be critical for monitoring norovirus evolution and informing public health response.

## Data Availability

The datasets for the 144 study isolates presented in this study can be found in the online repositories. The names of the repository/repositories and accession number(s) can be found at: https://www.ncbi.nlm.nih.gov/genbank/, PX999463–PX999606.
