# Supplementary material for: Global resurgence of norovirus GII.17[P17] accompanies shifting phylogeographic connectivity patterns: surveillance in Jiangsu, 2023–2025
Source: Front Microbiol. 2026 Oct 5;17:1861818. doi: 10.3389/fmicb.2026.1861818 (PMC13679624; doi:10.3389/fmicb.2026.1861818)
Supplement: Supplementary file 1 [file Image_1.pdf]

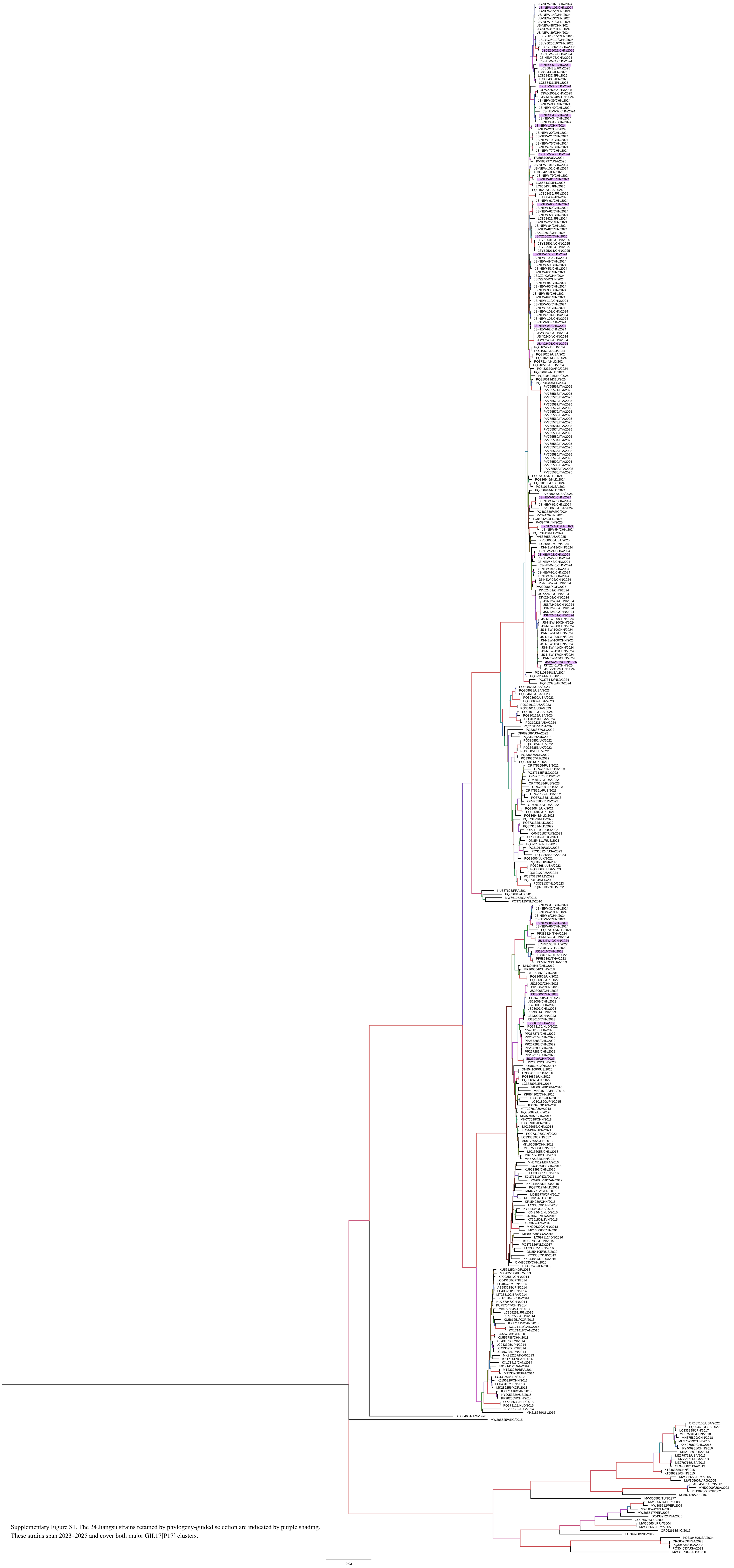

Supplementary Figure S1. The 24 Jiangsu strains retained by phylogeny-guided selection are indicated by purple shading. These strains span 2023–2025 and cover both major GII.17[P17] clusters.
