# Supplementary material for: Global resurgence of norovirus GII.17[P17] accompanies shifting phylogeographic connectivity patterns: surveillance in Jiangsu, 2023–2025
Source: Front Microbiol. 2026 Oct 5;17:1861818. doi: 10.3389/fmicb.2026.1861818 (PMC13679624; doi:10.3389/fmicb.2026.1861818)
Supplement: Supplementary file 2 [file Image_2.pdf]

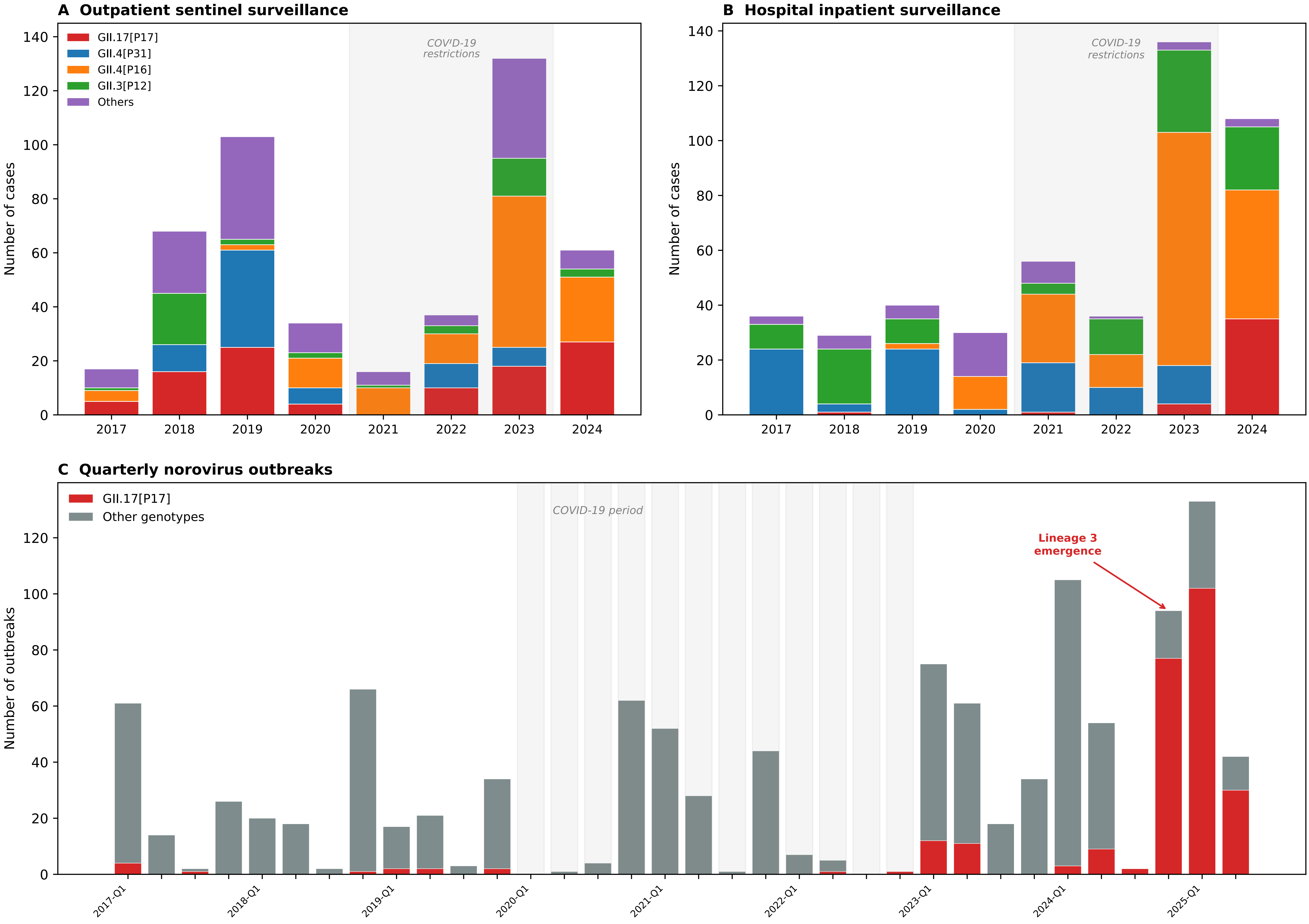

Supplementary Figure S2. Genotype replacement dynamics of norovirus in Jiangsu, 2017-2025. (A) Annual genotype composition from outpatient sentinel surveillance (2017-2024). (B) Annual genotype composition from hospital inpatient surveillance (2017-2024). The COVID-19 restrictions period (2020-2022) is shaded. (C) Quarterly norovirus outbreak timeline (2017 Q1-2025 Q2). The emergence of Lineage 3 in October 2024 is temporally coincident with the sharp inflection in outbreak counts.
