# Supplementary material for: Global resurgence of norovirus GII.17[P17] accompanies shifting phylogeographic connectivity patterns: surveillance in Jiangsu, 2023–2025
Source: Front Microbiol. 2026 Oct 5;17:1861818. doi: 10.3389/fmicb.2026.1861818 (PMC13679624; doi:10.3389/fmicb.2026.1861818)
Supplement: Supplementary file 3 [file Image_3.pdf]

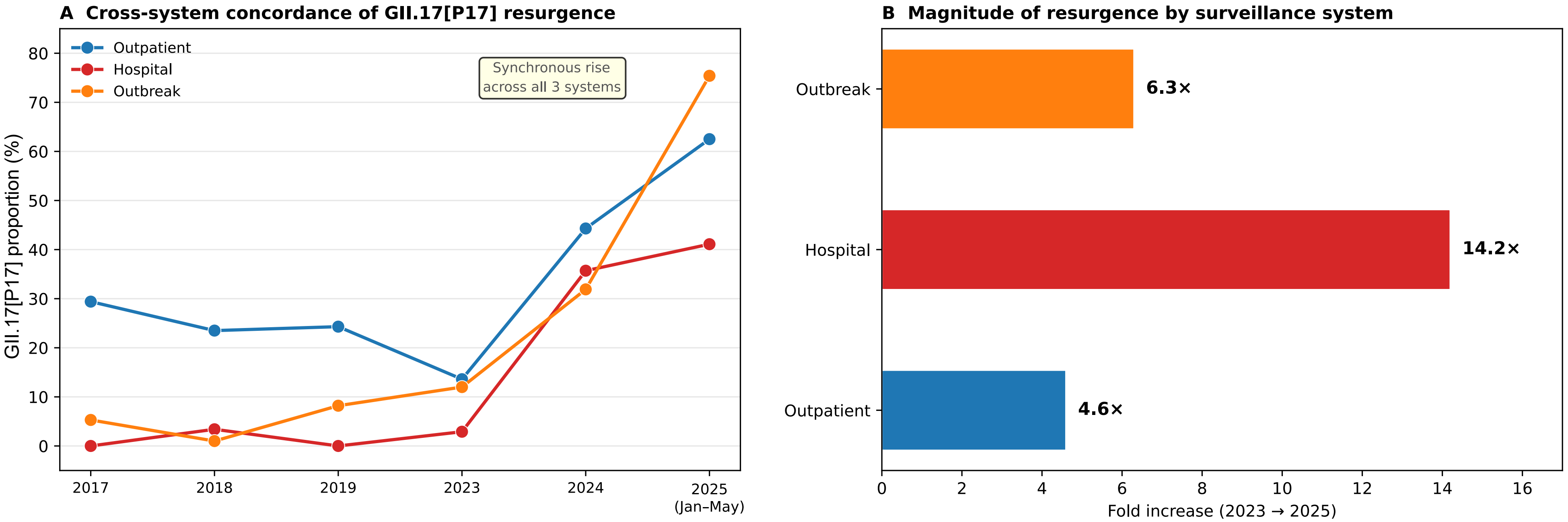

Supplementary Figure S3. Cross-system concordance of GII.17[P17] resurgence, Jiangsu, 2017-2025. (A) The proportions of GII.17[P17] among typed norovirus-positive specimens across three independent surveillance systems: outpatient sentinel, hospital inpatient, and outbreak. The synchronous rise across all three systems (2024-2025) confirms the resurgence is a genuine epidemiological phenomenon. (B) Fold increase in GII.17[P17] detection from 2023 to 2025 (January-May) by surveillance system.
