# Supplementary material for: Global resurgence of norovirus GII.17[P17] accompanies shifting phylogeographic connectivity patterns: surveillance in Jiangsu, 2023–2025
Source: Front Microbiol. 2026 Oct 5;17:1861818. doi: 10.3389/fmicb.2026.1861818 (PMC13679624; doi:10.3389/fmicb.2026.1861818)
Supplement: Supplementary file 4 [file Image_4.pdf]

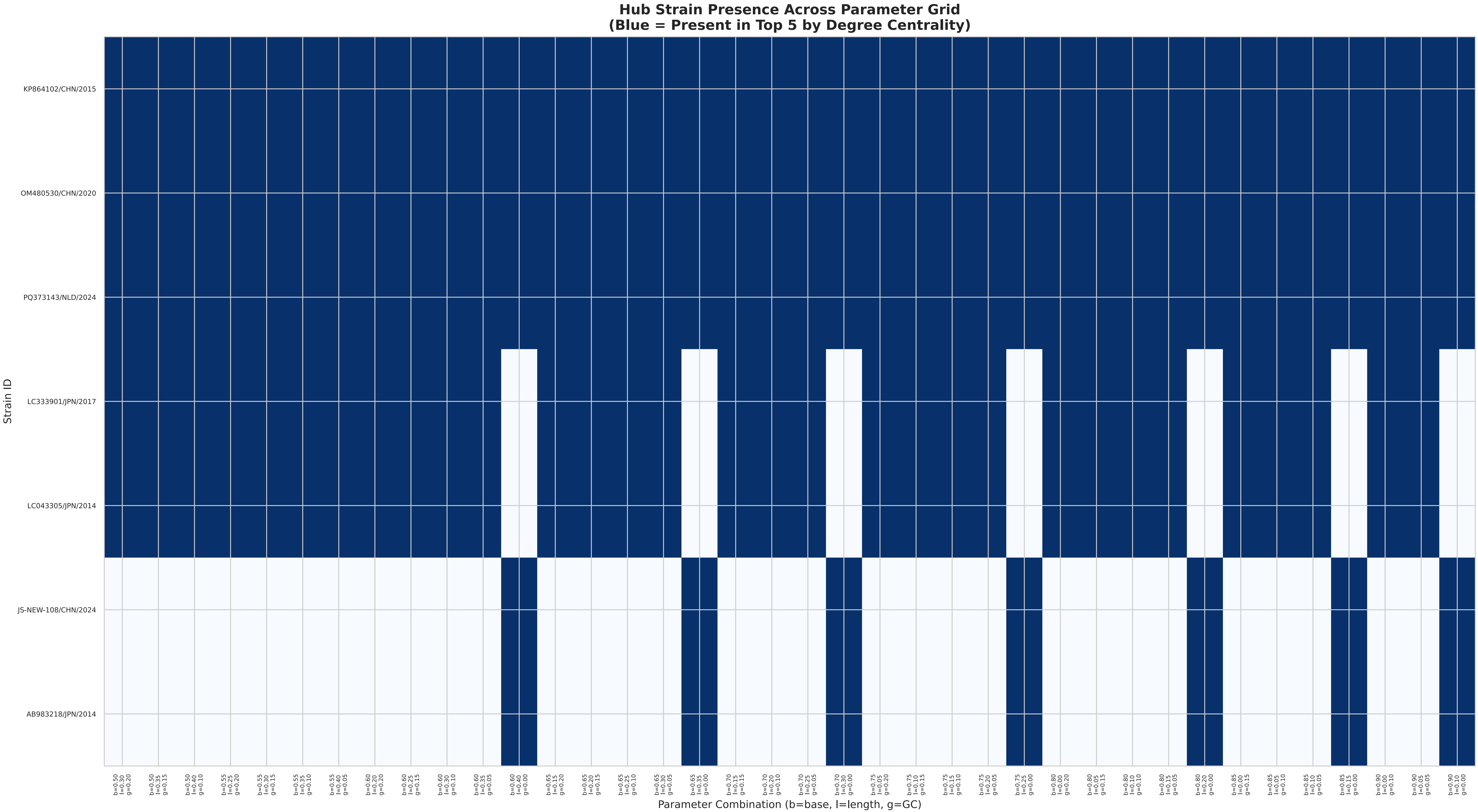

Supplementary Figure S4. Parameter sensitivity of minimum spanning tree (MST) hub identification. Heatmap showing hub consistency across 38 combinations of three distance-matrix weights: w_base (genetic p-distance, 0.5-0.9), w_len (length penalty, 0-0.4), w_gc (GC difference, 0-0.2). Colors encode the identity of the top three degree-centrality hubs. The three core hub strains (KP864102/CHN/2015, OM480530/CHN/2020, PQ373143/NLD/2024) were identified in 100% of all 38 parameter combinations.
