# Supplementary material for: Global resurgence of norovirus GII.17[P17] accompanies shifting phylogeographic connectivity patterns: surveillance in Jiangsu, 2023–2025
Source: Front Microbiol. 2026 Oct 5;17:1861818. doi: 10.3389/fmicb.2026.1861818 (PMC13679624; doi:10.3389/fmicb.2026.1861818)
Supplement: Supplementary file 5 [file Image_5.pdf]

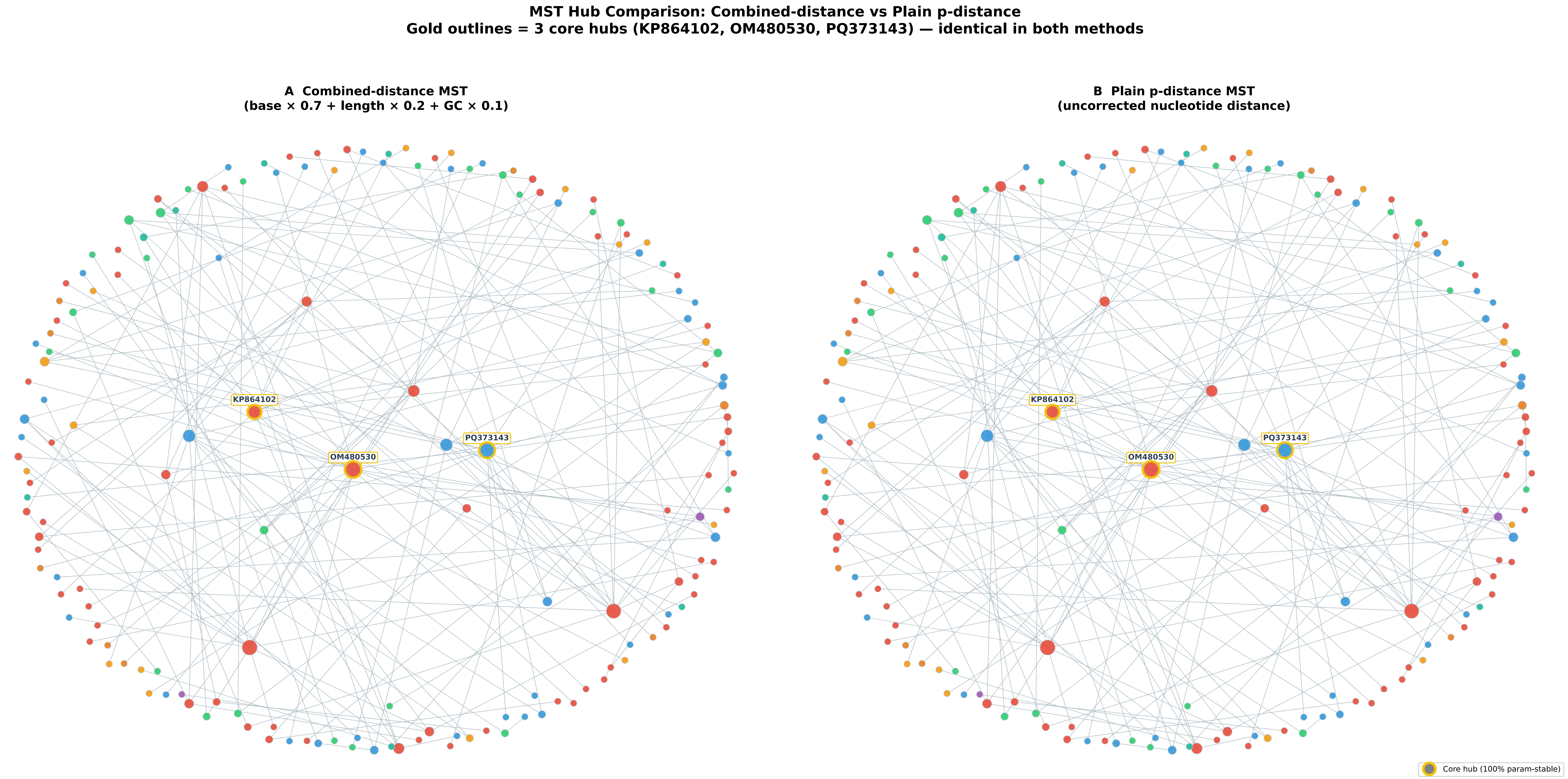

Supplementary Figure S5. Comparison of hub strain identification across network construction methods and centrality metrics. (A) Degree centrality under three network construction methods: MST (reference), threshold-based network (p10), and k-nearest neighbors (k = 5). (B) Hub identification by five independent centrality metrics (Degree, Betweenness, Closeness, Eigenvector, and PageRank). The three core hub strains appear in 2 of 5 metrics, providing convergent evidence for their network centrality.
