# Supplementary material for: Global resurgence of norovirus GII.17[P17] accompanies shifting phylogeographic connectivity patterns: surveillance in Jiangsu, 2023–2025
Source: Front Microbiol. 2026 Oct 5;17:1861818. doi: 10.3389/fmicb.2026.1861818 (PMC13679624; doi:10.3389/fmicb.2026.1861818)
Supplement: Supplementary file 6 [file Image_6.pdf]

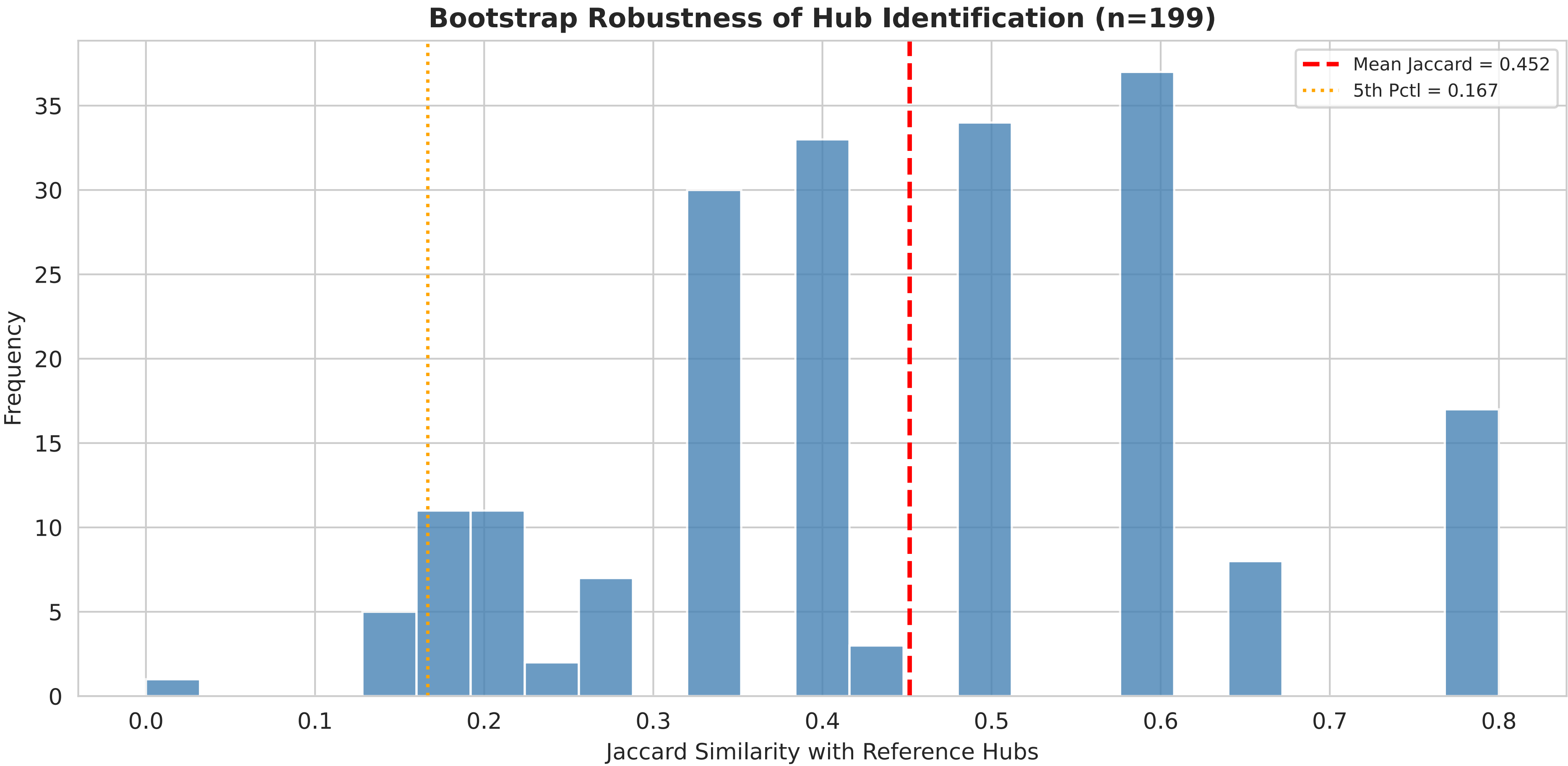

Supplementary Figure S6. Bootstrap resampling validation (200 iterations, 90% sequences sampled with replacement). Distribution of Jaccard similarity between full-dataset hub sets and bootstrap-resampled hub sets. Mean similarity = 0.452 ± 0.179 (SD). Individual hub recovery rates: OM480530/CHN/2020: 60.5%, PQ373143/NLD/2024: 56.0%, KP864102/CHN/2015: 53.5%.
