# Supplementary material for: Global resurgence of norovirus GII.17[P17] accompanies shifting phylogeographic connectivity patterns: surveillance in Jiangsu, 2023–2025
Source: Front Microbiol. 2026 Oct 5;17:1861818. doi: 10.3389/fmicb.2026.1861818 (PMC13679624; doi:10.3389/fmicb.2026.1861818)
Supplement: Supplementary file 7 [file Image_7.pdf]

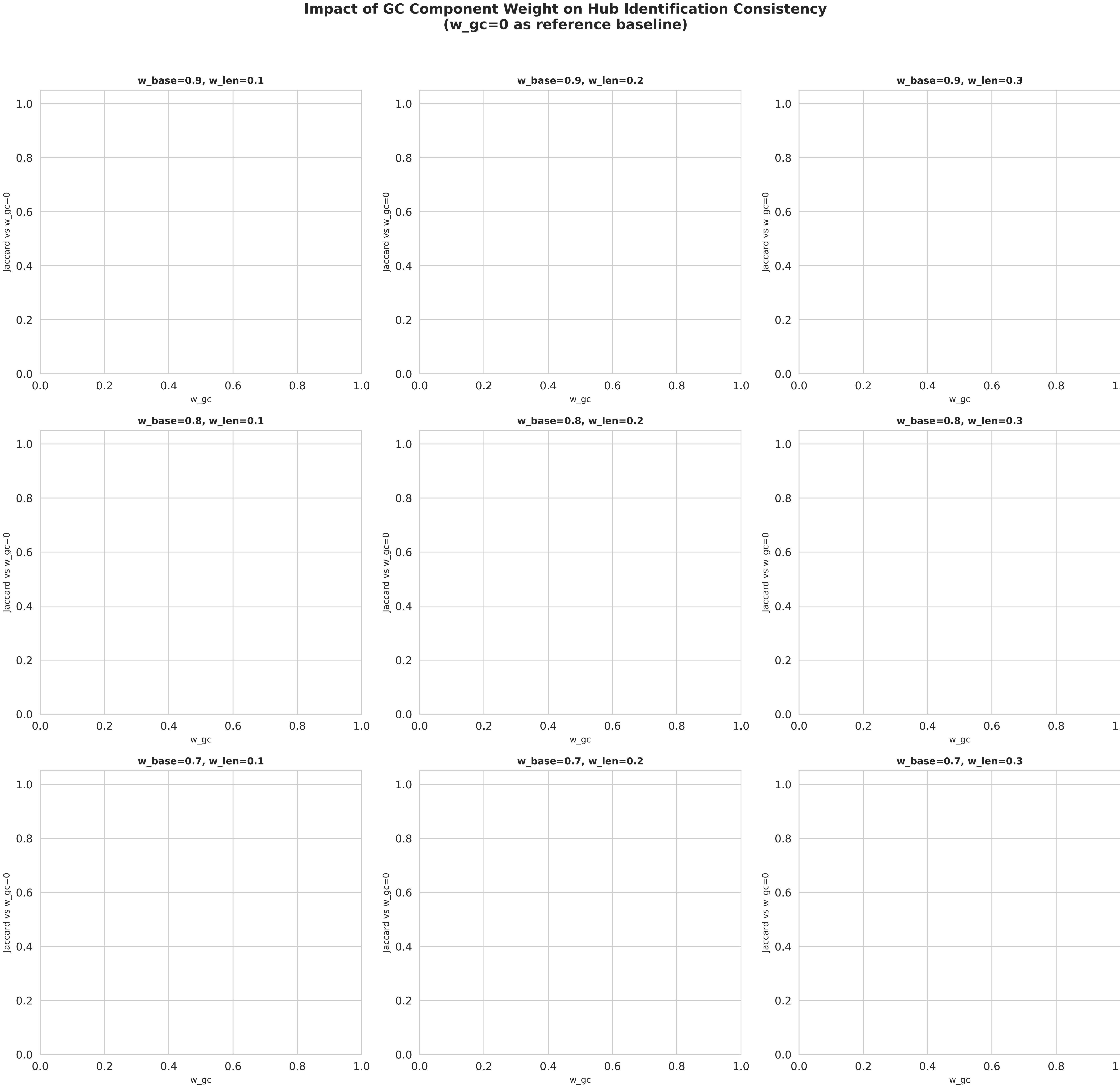

Supplementary Figure S7. Impact of GC weight (w_gc) on hub identification. Jaccard similarity of hub sets compared to baseline (w_gc = 0) across GC weight values of 0 to 0.2. Hub identification remains stable (Jaccard > 0.8 on all tested combinations), confirming that the GC component has negligible practical effect on hub inference in this dataset.
