# Supplementary material for: Global resurgence of norovirus GII.17[P17] accompanies shifting phylogeographic connectivity patterns: surveillance in Jiangsu, 2023–2025
Source: Front Microbiol. 2026 Oct 5;17:1861818. doi: 10.3389/fmicb.2026.1861818 (PMC13679624; doi:10.3389/fmicb.2026.1861818)
Supplement: Supplementary file 8 [file Image_8.pdf]

Adjusted Bayes Factor (BF_adj) Validation of Phylogeographic Inference
BSSVS with 7 continental regions | 6 subsampling strategies × 20M MCMC tip-state-swap

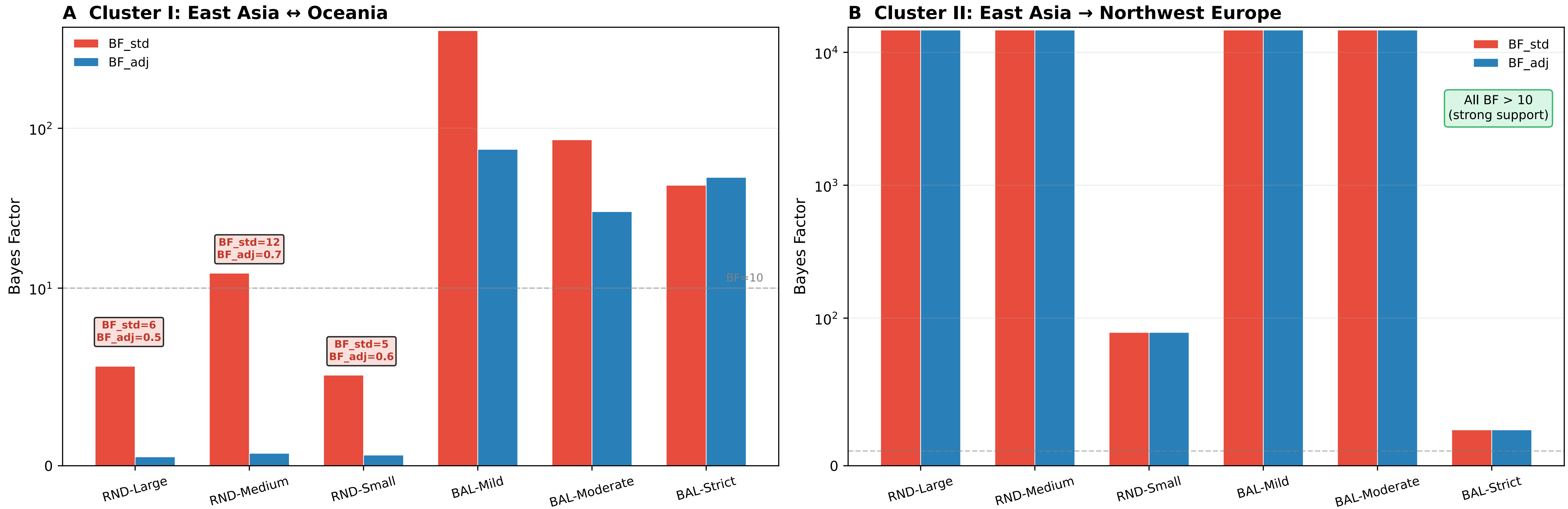

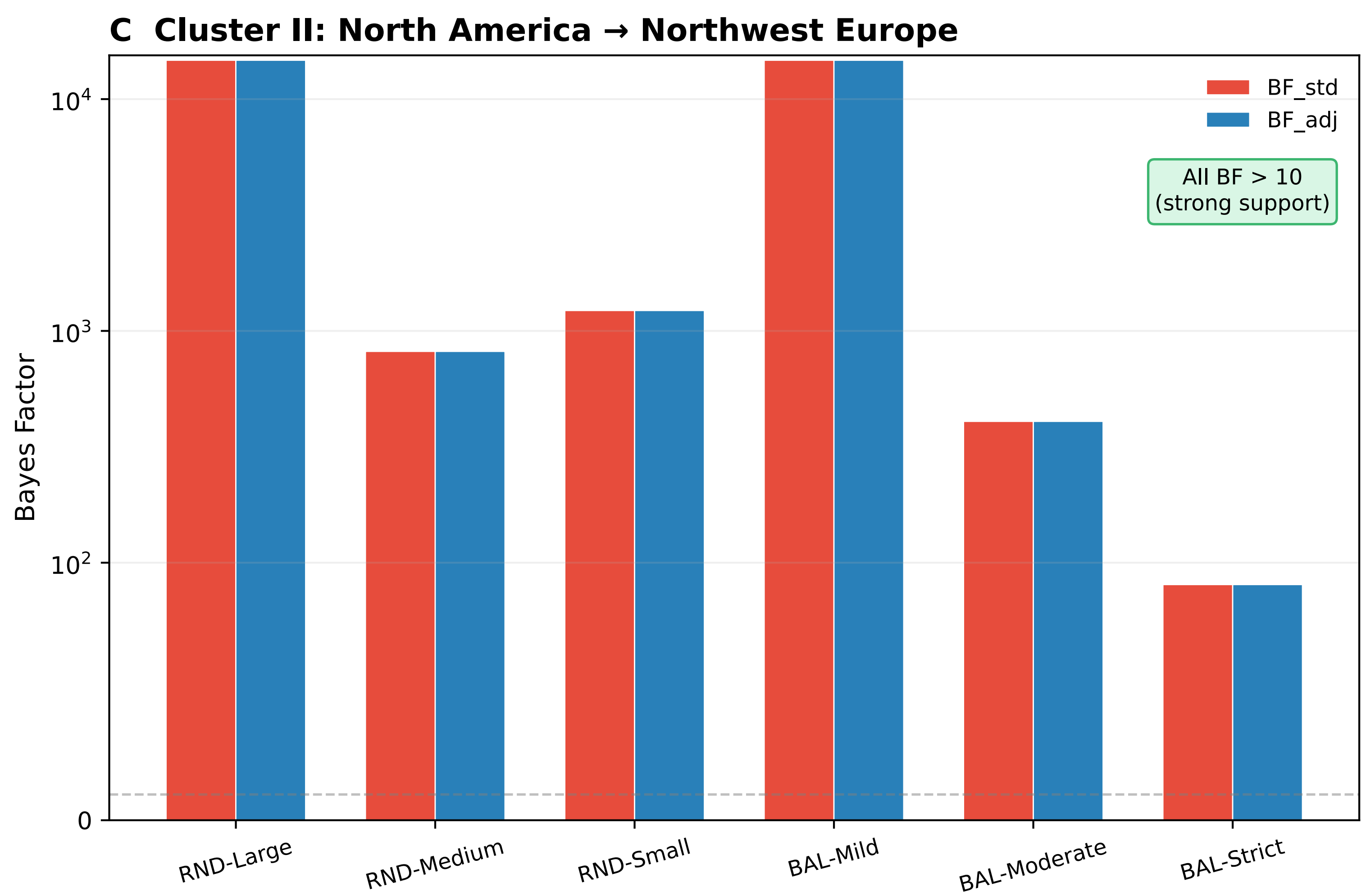

D Summary

	Cluster I	Cluster II
Key Link	East Asia ↔ Oceania	EA/NA → Northwest Europe
BF_std Range	5.1 – 408.1	24.3 – 14,712
BF_adj Range	0.5 – 73.8	24.3 – 14,712
Assessment	RND: ARTIFACT (BF_adj<1)<br>BAL: genuine, reduced 2-5×	Robust (6/6 strategies)<br>Stable under BF_adj

Supplementary Figure S8. Adjusted Bayes factor (BF adi) validation of phylogeographic inference across six subsampling strategies. (A) Cluster I: East Asia Oceania. BF Std (red) Vs. BF adj (blue, from tip-state-swap randomization. Under RND strategies (2 Oceania sequences). BF adj collapses to <1, confirming sparse-sampling artifact. Under BAL strategies (9 sequences) BF adj = 30-74, reflecting a modest genuine signal. Dashed line: BF = 10 (strong support). (B) Cluster II: East Asia Northwest Europe. BF std and BF adj are concordant; all six strategies exceed BF > 10, including BAL-Strict. (C) Cluster II: North America - Northwest Europe. Same pattern as panel B. (D) Summary of BF std and BF adj ranges and assessment.
