# Supplementary material for: Global resurgence of norovirus GII.17[P17] accompanies shifting phylogeographic connectivity patterns: surveillance in Jiangsu, 2023–2025
Source: Front Microbiol. 2026 Oct 5;17:1861818. doi: 10.3389/fmicb.2026.1861818 (PMC13679624; doi:10.3389/fmicb.2026.1861818)
Supplement: Supplementary file 9 [file Table_2.docx]

| Metric | Value |
| --- | --- |
| Total Jiangsu GII.17[P17] strains sequenced | 144 |
| Retained for phylogenetic analysis | 24 (16.7%) |
| Excluded | 120 (83.3%) |
| Retained by year | 2023: 4 \| 2024: 17 \| 2025: 3 |
| Excluded by year | 2023: 10 \| 2024: 99 \| 2025: 11 |

**Supplementary Table S2: Jiangsu 24-Strain Phylogeny-Guided Selection**

**Table S2-1. Data Overview**

## Table S2-2. Clustering Analysis — Excluded Strains Assigned to Closest Retained Strain

| Cluster (Retained Strain) | Excluded Strains | Mean Pairwise Identity | Min Identity | Max Identity |
| --- | --- | --- | --- | --- |
| Cluster 1: JS23010 | 100 | 0.9219 | 0.9185 | 0.9275 |
| Cluster 2: JS23006 | 7 | 0.9985 | 0.9975 | 1.0000 |
| Cluster 3: JS-NEW-85 | 6 | 0.9981 | 0.9975 | 1.0000 |
| Cluster 4: JS23015 | 6 | 0.9467 | 0.9460 | 0.9475 |
| Cluster 5: JS-NEW-9 | 1 | 1.0000 | 1.0000 | 1.0000 |
| Overall (120 excluded strains) | 120 | 0.9321 | 0.9185 | 1.0000 |

Median pairwise identity: 0.9210

Excluded strains with >99% identity to retained representative: 14.2% (17/120)

Excluded strains with >99.5% identity: 14.2%

## Table S2-3. Phylogenetic Diversity — Rarefaction Analysis

50 random subsamples per sample size; phylogenetic diversity = mean pairwise nucleotide distance

| Sample Size (n) | Mean Diversity | % of Full Diversity | Notes |
| --- | --- | --- | --- |
| 1 | 0.000000 | 0.0% | No pairwise diversity |
| 2 | 0.028259 | 101.4% | — |
| 3 | 0.031615 | 113.5% | — |
| 4 | 0.027744 | 99.6% | — |
| 5 | 0.027698 | 99.4% | — |
| 6 | 0.025726 | 92.3% | — |
| 8 | 0.027129 | 97.4% | — |
| 10 | 0.029665 | 106.5% | — |
| 12 | 0.029099 | 104.4% | — |
| 15 | 0.025156 | 90.3% | — |
| 18 | 0.029486 | 105.8% | — |
| 20 | 0.029535 | 106.0% | — |
| 24 (selected) | 0.038004 | 136.4% | Phylogeny-guided selection |
| 144 (full set) | 0.027863 | 100.0% | All 144 Jiangsu strains |

*Notes: Of the 144 Jiangsu GII.17[P17] strains sequenced (2023–2025), 24 were retained for phylogenetic analysis through a phylogeny-guided selection procedure. The excluded 120 strains clustered into five groups around their closest retained representative (Table S2-2). The 24-strain selection captures 136.4% of the total phylogenetic branch-length diversity of the 144-strain set — exceeding 100% because the excluded strains are concentrated in dense phylogenetic clusters (predominantly a 100-strain cluster centered on JS23010) that contribute minimal additional branch-length diversity. Mean pairwise nucleotide identity within each cluster is ≥92% for the large cluster and ≥99.8% for the four smaller clusters, confirming near-identity of excluded strains to their retained representatives. The final 24 strains span all three sampling years (2023: 4; 2024: 17; 2025: 3) and both GII.17[P17] clusters (CI-Lineage 2 and CII-Lineage 3).*
