# Supplementary material for: Global resurgence of norovirus GII.17[P17] accompanies shifting phylogeographic connectivity patterns: surveillance in Jiangsu, 2023–2025
Source: Front Microbiol. 2026 Oct 5;17:1861818. doi: 10.3389/fmicb.2026.1861818 (PMC13679624; doi:10.3389/fmicb.2026.1861818)
Supplement: Supplementary file 10 [file Table_6.docx]

# Supplementary Table S6: GII.17 VP1 Selection Pressure Analysis (HyPhy MEME/FEL/SLAC)

## A. MEME Episodic Positive Selection Sites (p < 0.1)

| **Alignment Codon** | **AB983218 AA Position** | **AB983218 Residue** | **LRT** | **p-value** |
| --- | --- | --- | --- | --- |
| 26 | 26 | T | 17.77 | <0.001 |
| 414 | 407 | E | 13.04 | <0.001 |
| 384 | GAP | — | 12.71 | <0.001 |
| 299 | 296 | R | 10.28 | 0.003 |
| 537 | 530 | N | 10.06 | 0.003 |
| 436 | 429 | F | 9.12 | 0.005 |
| 356 | 353 | V | 8.28 | 0.007 |
| 305 | 302 | Q | 6.79 | 0.015 |
| 379 | 375 | N | 6.47 | 0.018 |
| 395 | 389 | N | 5.34 | 0.032 |
| 22 | 22 | G | 5.15 | 0.035 |
| 347 | 344 | P | 5.00 | 0.038 |
| 256 | 256 | V | 4.49 | 0.049 |
| 8 | 8 | A | 3.43 | 0.085 |
| 382 | 378 | D | 3.40 | 0.086 |

*Note: Codon 384 corresponds to a gap in AB983218 and could not be mapped. Convergent evolution (10 independent branches) was detected at codon 299 (AB983218 position 296).*

## B. FEL Pervasive Positive Selection Sites (p < 0.1)

| **Alignment Codon** | **AB983218 AA Position** | **AB983218 Residue** | **p-value** |
| --- | --- | --- | --- |
| 21 | 21 | E | 0.052 |
| 298 | 295 | H | 0.098 |
| 435 | 428 | L | 0.008 |

## C. Lineage 3-Specific Mutations: Selection Pressure Status

| **AB983218 Position** | **Mutation (Ref→L3)** | **L3 Frequency** | **Functional Domain** | **MEME (p)** | **FEL (p)** |
| --- | --- | --- | --- | --- | --- |
| 350 | A→S | 94% | RBS-I | n.s. | n.s. |
| 361 | Q→R | 98% | P2 region (K361R) | n.s. | n.s. |
| 372 | R→K | 98% | Epitope A | n.s. | n.s. |
| 395 | D→N | 86% | Epitope D | n.s. | n.s. |
| 396 | H→Q | 86% | Adjacent to Epitope D | n.s. | n.s. |
| 469 | D→E | 86% | P1.2 region | n.s. | n.s. |

*Notes: Codon-level selection pressure analysis was performed on 192 GII.17 VP1 sequences (543 codons, aligned to reference strain AB983218/Kawasaki323/2014/JPN) using three complementary methods in HyPhy v2.5 (Pond et al., 2005). MEME (Mixed Effects Model of Evolution) detects episodic diversifying selection on individual branches; FEL (Fixed Effects Likelihood) estimates pervasive site-wise selection pressure under constant selective pressure; SLAC (Single-Likelihood Ancestor Counting) provides conservative verification. The marked discrepancy between MEME (15 episodic sites) and FEL (3 pervasive sites) / SLAC (0 sites) is the expected signature of episodic positive selection in an acutely infecting RNA virus. Position 296 in the P2 hypervariable loop showed convergent evolution on 10 independent branches (MEME, p = 0.003), strongly suggestive of immune-driven diversifying selection. For position 361 (K361R), despite the absence of codon-level positive selection signals, independent experimental validation by Tohma et al. (2025, Nat Commun) demonstrated enhanced Lewis a/Lewis x antigen binding, expanding host tropism to non-secretor individuals. The three-tier mutation classification (Critical Fixed / Fixed, Lineage-Specific / Sporadic) is detailed in Supplementary Table S5. References: Pond SLK, Frost SDW, Muse SV. (2005) Bioinformatics 21:676–679; Murrell B et al. (2012) PLoS Genet 8:e1002764; Kosakovsky Pond SL, Frost SDW. (2005) Mol Biol Evol 22:1208–1222; Tohma K et al. (2025) Nat Commun 16:11596.*
